# Determining biomolecular structures near room temperature using X-ray crystallography: concepts, methods and future optimization

**DOI:** 10.1107/S2059798322011652

**Published:** 2023-01-01

**Authors:** Robert E. Thorne

**Affiliations:** a Physics Department, Cornell University, Ithaca, NY 14853, USA; b MiTeGen LLC, PO Box 3867, Ithaca, NY 14850, USA; King’s College London, United Kingdom; University of Padova, Italy

**Keywords:** room-temperature crystallography, multi-temperature crystallography, radiation damage, serial crystallography, conformational heterogeneity

## Abstract

For roughly two decades, cryocrystallography has been the overwhelmingly dominant method for determining high-resolution biomolecular structures. Competition from single-particle cryo-electron microscopy and micro-electron diffraction, increased interest in functionally relevant information that may be missing or corrupted in structures determined at cryogenic temperature, and interest in time-resolved studies of the biomolecular response to chemical and optical stimuli have driven renewed interest in data collection at room temperature and, more generally, at temperatures from the protein–solvent glass transition near 200 K to ∼350 K. Fischer has recently reviewed practical methods for room-temperature data collection and analysis [Fischer (2021 ▸), *Q. Rev. Biophys.* **54**, e1]. Here, the key advantages and physical principles of, and methods for, crystallographic data collection at noncryogenic temperatures and some factors relevant to interpreting the resulting data are discussed. For room-temperature data collection to realize its potential within the structural biology toolkit, streamlined and standardized methods for delivering crystals prepared in the home laboratory to the synchrotron and for automated handling and data collection, similar to those for cryocrystallography, should be implemented.

## Abstract 2

Key physical principles and methods for collecting crystallographic data from biomolecular systems at room temperature and, more generally, at temperatures between ∼200 and ∼350 K are reviewed and discussed.

## 1. Introduction

Until synchrotron X-ray sources became available in the 1980s and 1990s, data collection in biomolecular crystallography was performed using large-diameter (>100 µm), weak and divergent X-ray beams from tube or rotating-anode sources and large (>100 µm) crystals mounted within glass X-ray capillaries and held at room temperature (Blundell & Johnson, 1976 ▸). Drawing a crystal into a fragile 10 µm-wall glass capillary, removing excess mother liquor from around the crystal to affix it to the capillary wall, adding a plug of mother liquor and sealing the capillary using wax to maintain crystal hydration, and then mounting the sealed capillary in the X-ray beam, required considerable skill. Crystallization drops were disrupted and crystals were damaged from interactions with the capillary; crystals dehydrated when wax seals were imperfect, or were swallowed up as changes in atmospheric pressure or temperature moved the mother-liquor plug within the capillary; capillaries snapped in clumsy handling; and crystals slipped and settled as the capillary was rotated. Data collection from each crystal using laboratory X-ray sources required exposures of tens of hours. Radiation damage often necessitated data collection from multiple crystals. Crystal dehydration during loading into capillaries, due to imperfect capillary seals and due to fluctuations in ambient temperature, resulted in substantial crystal non-isomorphism, degrading data-set quality.

The development of cryocrystallographic methods in the 1990s and 2000s (Garman & Schneider, 1997 ▸; Rupp, 2009 ▸; Pflugrath, 2015 ▸; based in part on methods used in cryo-electron microscopy and in cell and tissue cryopreservation), together with the increasing availability of crystallography beamlines at synchrotron X-ray sources, transformed bio­molecular crystallography. Crystals can easily be harvested from drops using nylon or microfabricated loops attached to standard goniometer bases and then immediately plunged into liquid nitrogen, with a reduced risk of crystal damage and dehydration during mounting. Samples held at 77 K are structurally stable and mechanically robust, allowing sample preparation long before data collection, easy storage and easy shipping. Cryocooled crystals cannot slip or dehydrate, and diffract far more photons before radiation damage becomes problematic, so fewer/smaller crystals are required to collect a complete data set. Initial reservations that cryocooling might strongly perturb protein conformation were largely unsupported by experiment, although relatively few direct comparisons of high-resolution structures determined from identically prepared crystals at room and cryogenic temperature were reported. By the mid-2000s nearly all crystallo­graphic structure determinations were performed using cryocooled crystals.

Several factors have driven renewed interest in crystallo­graphic data collection at and near room temperature. These include development of X-ray free-electron laser (XFEL) sources, of serial sample-delivery methods required for their efficient utilization and of software to process and model the diffraction patterns collected from enormous numbers of small crystals (Barends *et al.*, 2022 ▸); extension of XFEL methods to data collection at synchrotron sources; an increased appreciation of (and improved methods for mining) biologically relevant conformational heterogeneity that may be perturbed by cryocooling (Fraser *et al.*, 2011 ▸); and new methods for time-resolved study of reactions and conformational dynamics within crystals (Brändén & Neutze, 2021 ▸).

A key enabler of expanded room-temperature studies has been the greatly increased ease of collecting and analyzing diffraction data from large numbers of crystals. In traditional macromolecular crystallography (MX), the usual goal is to mount a single crystal on each support (for example a loop), so data collection from many crystals requires serial handling of many supports. ‘High-throughput’ (HT) crystallography methods developed in the first decade of this century made this especially easy for cryogenically cooled crystals, but data collection from crystals at room temperature remained awkward. In the ‘serial crystallography’ (SX) methods developed in the second decade of this century, many crystals can be mounted on a single support (a ‘fixed target’) and the crystals serially translated via step-and-repeat or continuous rastering into a small X-ray beam. Alternatively, crystals may be embedded in a liquid or gel stream or dispensed onto a moving ‘tape’ and serially translated into the X-ray beam. These approaches address the gaps in sample-handling ease and efficiency between room and cryogenic temperature.

Fischer (2021 ▸) has recently provided a comprehensive review of methods in room-temperature crystallography. The present review is intended as a complement, focusing upon advantages, physical aspects and key challenges, and upon advances that address these challenges. Reviews of serial room-temperature crystallography (Grünbein & Kovacs, 2019 ▸; Martiel *et al.*, 2019 ▸; Cheng, 2020 ▸) have emphasized applications involving data collection from enormous numbers (10^4^–10^6^) of similar-sized crystals and that may require the production of >10^9^ crystals. The present review focuses on less technically ambitious versions of serial crystallography that are better suited to the far more common situation in which crystals generated in routine screening and optimization are more modestly abundant and are heterogeneous in size and morphology. This review does not address time-resolved crystallography, as the key additional issues there, involving reaction initiation and its timing before X-ray data collection, are largely orthogonal to those discussed here. The review concludes with suggestions for what is needed for widespread application of room-temperature crystallography by the structural biology community.

## 2. Why collect data at room temperature?

### 2.1. Probing conformational ensembles

The overwhelming majority of structural models obtained from cryocrystallographic data are refined to a single conformation. Additional electron-density features are observable in both cryogenic and especially room-temperature maps (Fraser *et al.*, 2009 ▸, 2011 ▸; Lang *et al.*, 2010 ▸, 2014 ▸; Keedy *et al.*, 2014 ▸, 2015 ▸). These features may correspond to alternative, usually lower-occupancy conformations that may play important roles in ligand binding, catalysis and allosteric regulation and thus are important for mechanistic understanding. These density features typically undergo substantial remodeling between room/biological temperature and ∼200 K (near the protein–solvent glass transition; Ringe & Petsko, 2003 ▸; Fenimore *et al.*, 2004 ▸; Lagi *et al.*, 2008 ▸; Doster, 2010 ▸) and then remain nearly constant on further cooling (Keedy *et al.*, 2015 ▸). Regions with ordered density at, for example, cryogenic temperature may be disordered at room temperature, and vice versa.

Conformational remodeling occurs for several reasons: unit cells contract more on cooling than protein volumes (Frauenfelder *et al.*, 1979 ▸, 1987 ▸; Quillin & Matthews, 2000 ▸; Juers & Matthews, 2001 ▸, 2004*b* ▸; Alcorn & Juers, 2010 ▸; Atakisi *et al.*, 2018 ▸), so some conformations are blocked or modified by tighter packing; interactions (for example those dependent on p*K*_a_ values) that drive local folds vary with temperature; and thermal occupation of higher energy conformations is reduced. Room/biological temperature data collection and, as discussed in Section 4.5, data collection at multiple temperatures between 200 and ∼350 K can facilitate the identification of the most important alternative conformations in active sites and elsewhere.

### 2.2. Cryoprotectants

The penetrating cryoprotectants used in cryocrystallography can perturb side-chain conformations. Those that are less strongly excluded (for example MPD, DMSO and ethylene glycol) may perturb the hydration water structure. Cryo­protectants can contribute density, including near active sites, that can obscure or be misidentified as low-occupancy ligands (Rupp, 2009 ▸). Cryoprotectants increase the average solvent electron density and thus reduce the electron-density contrast between biomolecule and solvent (Tyree *et al.*, 2018 ▸), reducing the Bragg peak intensities relative to the diffuse X-ray background (Supplementary Fig. S8). All of these effects are overcome by collecting room-temperature data using crystals grown or soaked in ‘clean’, cryoprotectant-free buffers. Other small molecules/ions used to promote crystallization can sometimes also be ‘flushed’ (soaked out) without short-term loss of crystal order or integrity, especially when small crystals (which are less likely to fracture) are used, allowing high-quality room-temperature diffraction data to be collected within some finite time period after flushing.

### 2.3. Avoiding cryocooling and cooling-induced crystal disorder

If cryoprotectant concentrations and/or cooling rates during plunge-cooling are inadequate, internal ice may form that will degrade the crystal order and the diffraction resolution (Rupp, 2009 ▸; Pflugrath, 2015 ▸; Moreau *et al.*, 2019*a* ▸, 2021 ▸). Ice diffraction from any source, including internal crystal ice, ice formed in solvent outside the crystal and frost (for example, picked up from liquid nitrogen used for cooling and storage or formed from moist ambient air in, for example, non-optimally adjusted cryostreams), contributes background diffraction that biases experimental structure factors near the ice-ring resolutions (Thorn *et al.*, 2017 ▸; Parkhurst *et al.*, 2017 ▸; Moreau *et al.*, 2021 ▸).

Cryocooling crystals always degrades the lattice order, even when the internal solvent vitrifies and even though thermal disorder (a contributor to atomic and Wilson *B* factors) is reduced (Juers & Matthews, 2004*a* ▸). Cryocooling can introduce substantial crystal non-isomorphism (Giordano *et al.*, 2012 ▸), which is of particular importance when crystals contain weak anomalous scatterers (Akey *et al.*, 2016 ▸). Well-ordered crystals at room temperature have much smaller mosaicities (∼0.01° or less; Shaikevitch & Kam, 1981 ▸; Dobrianov *et al.*, 1999 ▸) than cryocooled crystals (∼0.2° or more) and much narrower distributions of unit-cell sizes within each crystal (Kriminski *et al.*, 2002 ▸). Increased crystal disorder at cryogenic temperature results from differences in the thermal contraction of the internal crystal solvent volume, protein volume and unit-cell volume, which drive inhomogeneous redistribution of solvent within the crystal and associated lattice disruptions (Juers & Matthews, 2001 ▸, 2004*a* ▸,*b* ▸; Kriminski *et al.*, 2002 ▸; Moreau *et al.*, 2019*b* ▸); from incomplete relaxation of protein and lattice structure towards their temperature-dependent equilibrium during cooling, leading to quenched heterogeneity (Moreau *et al.*, 2019*b* ▸); and from crystal bending and cracking caused by external and internal stresses during cryocooling. Cold crystal unit cells and order also depend on the cooling rates (Moreau *et al.*, 2019*b* ▸), which are poorly controlled and highly variable in current cryocrystallography practice. Variability in cryo­protectant soaks and cooling can also cause substantial cold crystal non-isomorphism.

All issues with ice and cooling-generated crystal disorder are avoided in room-temperature data collection. Cryogenic temperature data collection typically gives higher resolution data sets, but this is in part due to freeze-out/blocking of alternative conformations during cooling, and thus a loss of possibly biologically relevant information, due to reduction of thermal atomic motions and ordering of additional hydration waters (which may be biologically irrelevant); and due to the fact that laboratory and synchrotron X-ray data collection setups have typically been optimized for cryogenic temperature data collection and the properties of cold crystals, which reduces the achievable Bragg diffraction signal to background and diffraction resolution at room temperature.

### 2.4. Troubleshooting sample preparation

Identifying cryoprotection conditions/protocols that maintain crystal order during both soaking and cooling, minimize osmotic shock and fracturing, and prevent ice formation inside and outside the crystal consumes both time and crystals, and can be particularly challenging for membrane proteins and for crystals grown in lipidic cubic phase (LCP) and gel phases. When poor cryogenic temperature diffraction is obtained, the reason, which may be damage caused by cryoprotectant soaks (from osmotic shock or conformation/lattice changes), damage caused by cryocooling or as-grown crystal disorder, is seldom obvious and usually cannot be diagnosed using cryocrystallographic data alone. Room-temperature data collection avoids these issues and provides a reliable diagnostic for disorder introduced by cryocrystallographic sample-preparation protocols.

### 2.5. Complementing single-particle cryo-EM

Competition from single-particle cryo-electron microscopy (cryo-EM) and emerging competition from micro-electron diffraction (micro-ED) are among several factors driving renewed interest in room-temperature X-ray crystallography. Advances in direct electron detectors, phase plates and analysis software have produced a ‘resolution revolution’ in cryo-EM (Vinothkumar & Henderson, 2016 ▸; Cheng, 2018 ▸; Glaeser, 2019 ▸). The fraction of protein structures released in the PDB each year determined by cryo-EM and by X-ray crystallo­graphy changed from ∼0.7% and 94%, respectively, in 2012 to 26% and 71% in 2021; the fraction of cryo-EM structures with resolutions <3 Å and >4.5 Å changed from 1.5% and 93%, respectively, in 2012 to 22% and 11% in 2021 (from https://www.rcsb.org/stats/all-released-structures). Cryo-EM requires only biomolecules in solution, requires (in favorable cases) only tiny amounts of biomolecules and can be attempted as soon as biomolecules are available. Cryo-EM can use full-length proteins, including flexible regions that may make crystallization challenging, and can capture (via freeze-quenching) proteins in multiple main-chain conformations (including those that may be inaccessible in available crystal forms).

However, cryo-EM has its own set of challenges. Protein particles in the initially deposited solution are strongly concentrated by blotting and evaporation. They are further concentrated by accumulation at the air–buffer and buffer–foil interfaces, where they may be preferentially oriented and/or partially denatured (Glaeser, 2018 ▸; Noble *et al.*, 2018 ▸) and where 2D aggregates may form. The fraction of solvent in particle-dense interfacial layers whose structure and dynamics are strongly perturbed by hydration and interface interactions may be comparable to the fraction of perturbed solvent within higher solvent-content protein crystals (Moreau *et al.*, 2019*a* ▸,*b* ▸). Main-chain conformational ensembles may be substantially perturbed, including in ways that may be atypical of crowded cellular environments. Cooling times in cryo-EM (>0.1 ms; Ravelli *et al.*, 2020 ▸; Engstrom *et al.*, 2021 ▸) are only modestly faster than the ∼1 ms achievable in cryocrystallography (Clinger *et al.*, 2021 ▸), and are likely to be too slow to reliably capture the room/biological temperature conformational ensembles of side-chain rotamers and perhaps also of smaller loops and flaps. Precooling by ∼30–50 K as cryo-EM samples traverse (in ∼1–2 ms) the few millimetres of cold gas that is invariably present above liquid ethane (Engstrom *et al.*, 2021 ▸) is likely to further perturb side-chain conformations.

Micro-electron diffraction allows biomolecular structures to be determined using only a small number of submicrometre-sized crystals and is feasible because the interaction of matter with electrons is orders of magnitude stronger than that with X-rays (Nguyen & Gonen, 2020 ▸). To minimize radiation damage and to prevent crystal dehydration in the vacuum of the electron microscope, samples must be cryocooled, imposing similar constraints as in X-ray cryocrystallography. Growing crystals that are thin enough to be adequately electron-transparent is challenging, so much larger crystals (often suitable for X-ray analysis) are ion-milled into lamella.

Room-temperature X-ray crystallography can yield complementary information to these electron-based methods. Cryo-EM has advantages for observing main-chain conformational heterogeneity (which is often quenched by crystal packing), and room/biological temperature crystallography has advantages for observing other heterogeneities salient to enzymatic function, to ligand interactions and to protein and ligand design. Cryo-EM can yield atomic resolution reconstructions (see, for example, Yip *et al.*, 2020 ▸; Gijsbers *et al.*, 2021 ▸), but resolutions are and are likely to remain lower than those in crystallography. In 2021, 92% of released protein crystal structures had resolutions <3 Å and 47% had ‘near-atomic’ (Wlodawer & Dauter, 2017 ▸) or atomic resolutions (<2 Å), compared with 22% and only 0.4%, respectively, of cryo-EM structures. High-resolution cryo-EM imaging is so far restricted to molecular weights above ∼100 kDa, which is larger than most proteins (for example, the median molecular weight of human proteins is ∼40 kDa), although this can be circumvented by forming complexes (see, for example, Wu & Rapoport, 2021 ▸) with a possible loss of conformational flexibility. High-resolution cryogenic and especially room-temperature X-ray crystallography will be needed to corroborate many cryo-EM findings, and in most cases to visualize side-chain conformations, water molecules and ligand interactions in active sites important in function. When crystals are available, near-atomic resolution structure-determination throughputs in, for example, ligand screening are and are likely to remain orders of magnitude greater in crystallo­graphy than in cryo-EM.

## 3. Challenges in room-temperature crystallography

Room-temperature data collection eliminates time-, crystal- and money-consuming steps of cryoprotectant soaks and cryocooling and their optimization; it eliminates deleterious effects on crystal order and diffraction associated with these steps; it simplifies the diagnosis of sample-quality issues; and it is more likely to reveal biologically relevant conformational heterogeneity and ligand-binding states. Why has it remained a poor second choice in the minds of most who use crystallography?

The key challenges in room-temperature crystallography are associated with radiation damage, with increased total crystal volumes required for structure determination, with maintaining crystals in their as-grown state through to the end of data collection, and with interpreting additional electron-density features visible in room-temperature maps. Table 1 ▸ briefly summarizes these challenges.

### 3.1. Radiation damage

Biomolecular crystals are much more sensitive to radiation damage at room temperature than at cryogenic temperature (Nave & Garman, 2005 ▸; Holton, 2009 ▸; Warkentin & Thorne, 2010*a* ▸; Warkentin *et al.*, 2017 ▸). Beyond this basic fact, many details of damage at room temperature relevant to optimizing data collection are incompletely characterized.

At cryogenic temperatures all protein crystals appear to be comparably radiation-sensitive, as measured by rates of decay of diffracted intensity within Bragg peaks with dose in Grays, regardless of sequence, chemical composition, solvent content and structure. The rate of decay with dose of integrated Bragg intensity within a given resolution shell varies roughly as the square of the inverse resolution. The maximum tolerable dose in data collection thus depends strongly on the desired or the available resolution. The half-dose (at which the integrated intensity drops by half) is roughly 15 MGy for a near-atomic resolution of ∼1.5 Å (Atakisi *et al.*, 2019 ▸), as proposed by Henderson (1990 ▸) based on experience in cryo-EM and first verified in X-ray crystallography by Teng & Moffat (2000 ▸, 2002 ▸).

At room temperature, measurements on crystals of several proteins including lysozyme have yielded half-doses of 200–600 kGy for resolutions of ∼2 Å (Nave & Garman, 2005 ▸; Leal *et al.*, 2013 ▸; Schubert *et al.*, 2016 ▸; Warkentin *et al.*, 2017 ▸; Gotthard *et al.*, 2019 ▸; de la Mora *et al.*, 2020 ▸), with values corrected for non-uniform crystal irradiation falling near 200 kGy (Warkentin *et al.*, 2017 ▸; Atakisi *et al.*, 2019 ▸), roughly 50 times smaller than at cryogenic temperature for the same protein at comparable resolution. However, room-temperature half-doses show much more variation with the protein crystal system. Anecdotal evidence and limited quantitative data from crystals with relatively poor resolution and large solvent contents/large solvent cavities (Leal *et al.*, 2013 ▸; Warkentin *et al.*, 2014 ▸) suggest that some crystals are up to 10^3^ times more sensitive (when comparing diffraction-spot fade-out at equal resolution) at room temperature.

At cryogenic temperatures, integrated Bragg intensities show near-exponential decays (after correction for the effects of non-uniform crystal irradiation) with dose (Atakisi *et al.*, 2019 ▸), and unit cells and mosaicity show linear increases with dose, both over a dose range larger than the half-dose *D*_1/2_. At room temperature, anecdotal results suggest that more complex variations with dose, for example where the rate of intensity decay increases at large doses, can be observed.

At cryogenic temperatures, the rates of site-specific radiation damage with dose, for example the breakage of disulfide bonds and the reduction of metal centers, can be one to two orders of magnitude larger than the global damage rate given by the overall fading of Bragg diffraction (Ravelli & McSweeney, 2000 ▸; Weik *et al.*, 2000 ▸; Meents *et al.*, 2007 ▸). However, available data suggest that at room temperature site-specific damage rates are much closer to those of global damage (Roedig *et al.*, 2016 ▸; Gotthard *et al.*, 2019 ▸; de la Mora *et al.*, 2020 ▸).

Unlike at cryogenic temperature, where vitrified internal crystal solvent forms a solid network and thermal motion of atom-sized and larger species is quenched, at room temperature radiolytic products diffuse and react, causing additional bond-scale damage; damaged side and main chains can change their conformation; and entire molecules may be displaced or rotated. Each damaged bond can thus result in displacement of a much larger number of atoms (Warkentin *et al.*, 2013 ▸), and these atomic displacements dominate in causing spot fading. A large variability in room-temperature radiation-sensitivity may be expected based upon the stability of the fold of a protein and upon the tightness of its packing within the crystal, which in turn may depend upon the number density of crystal contacts, the crystal solvent content and solvent-cavity sizes, and macroscopic crystal properties such as yield strength. Reduced differences between site-specific and global damage rates at room temperature are consistent with the diffusion and reaction of free radicals and the motion of groups of atoms in response to bond-scale damage becoming the dominant source of spot fading. Global rates of damage with dose are increased towards those of specific sensitive sites, and ‘sensitive’ sites undergo more spot-fading displacements due to the same processes that affect other regions of the protein.

Quantitative sampling of room-temperature radiation-damage sensitivities in diverse crystal systems is needed to identify biomolecule and crystal parameters that correlate with radiation-sensitivity and to allow the robust prediction of maximum tolerable doses in data collection. This sampling should use standard metrics (for example half-doses for the overall diffraction pattern and for the integrated intensity in each resolution shell) and measurement protocols (for example flat-top beams and repeated oscillation over a small angular wedge) to facilitate quantitative comparison. A one-click beamline-control routine that automatically acquires the required diffraction data and applies required scale factors to generate quantitative radiation-sensitivity estimates would facilitate the compilation of these data for predicting dose limits and optimizing data collection.

### 3.2. Sample-volume requirements

Because of increased radiation-sensitivity, structure determination (when using non-XFEL sources and dose rates of <10 MGy s^−1^; see Section 4.1) at room temperature requires more total crystal volume (roughly 50×) and/or larger crystals (roughly 4× in linear dimension) than at cryogenic temperatures. However, this overstates the room-temperature penalty. It does not count the crystals consumed in identifying and optimizing cryoprotection conditions, including vain attempts to improve diffraction quality when the as-grown crystal order is inadequate. As emphasized by Fischer (2021 ▸), individual crystals of ordinary size are often sufficient to obtain complete room-temperature data sets for ordinary-sized proteins. Formal analysis assuming spherical crystals, data collection to 2 Å resolution and a maximum dose 50× smaller than at *T* = 100 K gives minimum crystal diameters of 15 and 31 µm for molecular weights of 10 and 100 kDa, respectively (Holton & Frankel, 2010 ▸). However, if only 5 µm crystals are available then (roughly) 26 and 230 crystals are required, respectively (compared with one and ∼5 at *T* = 100 K); if the molecular weight is 1 MDa, then one 66 µm crystal or ∼2300 5 µm crystals are required. In cases of small crystals and/or large molecular weights, optimizing all aspects of room-temperature sample preparation and data collection to maximize Bragg diffraction signal to background and crystal isomorphism can have a substantial payoff.

### 3.3. Crystal dehydration and non-isomorphism

Since many more crystals may be required for structure determination at room temperature, crystal non-isomorphism can be a more serious issue than at cryogenic temperature, particularly if a goal is to observe and model weak electron-density features.

Dehydration is the dominant cause of crystal non-isomorphism in room-temperature data collection and is a major contributor at cryogenic temperatures (Farley *et al.*, 2014 ▸). Modest dehydration (to, for example, 93% relative humidity) can cause unit-cell shrinkages, crystal-packing increases, depopulation of alternative conformers and remodeling of side chains comparable in magnitude and extent to those of cryocooling (Atakisi *et al.*, 2018 ▸). The extent of dehydration can vary substantially from crystal to crystal on the same loop/support (depending on the crystal size and the extent of coverage by solvent or oil) and especially between crystals on different supports.

Crystal dehydration can occur while a crystal is still in its crystallization drop, once it is exposed to air for crystal harvesting; during crystal soaks in drops or wells exposed to air; during mounting on a sample loop/support, before the support is sealed; and after mounting and sealing and through to the end of data collection, due to water-vapor absorption by and transmission through sample-mount materials and due to improper seals. The risk of dehydration is greatest when crystals are harvested from the small-volume (200–600 nl) drops used in high-throughput screening and when the crystals are small (<50 µm). At 21°C and 50% relative humidity (r.h.) water drops of volumes 2 µl, 200 nl, 20 nl and 2 nl lose 10% of their volume in ∼200, 45, 10 and 2 s, respectively, with the required time decreasing by roughly a factor of five for each factor of ten decrease in the initial drop volume (Carrier *et al.*, 2016 ▸). Since 2 nl is the volume of a 150 µm diameter drop, the challenge of keeping sub-100 µm and especially sub-10 µm crystals fully hydrated, even accounting for the somewhat slower evaporation of solvent from the crystal interior, is clear.

Evaporation rates from a protein drop or crystal scale roughly linearly with (*x*_s_ − *x*), where *x* is the mass of water vapor per mass of dry air at ambient relative humidity and *x*_s_ is the mass ratio for air with a water activity corresponding to that of the protein drop/crystal. Since typical water activities in protein crystals and buffer solutions correspond to ≥97% r.h. (particularly when PEG is used as a precipitant; Èliassi *et al.*, 1999 ▸), increasing the ambient humidity from 50% to 90% and 98% increases the working times by factors of five and >25, respectively. Working in a cold room at 4°C (when crystallization trays and crystals tolerate it) reduces evaporation rates and increases working times at a given r.h. by a more modest factor of ∼3.

Aside from dehydration, crystal non-isomorphism can arise in several other ways. In most crystallization approaches, water leaves the protein-containing drop over time, either by design (as in vapor-diffusion growth) or because of limitations of the crystallization device materials (for example the finite water-absorption and finite water-vapor transmission rates of polymer crystallization trays and sealing films). As a result, the water activity/hydration of a given crystal will depend on when it is harvested relative to when the crystallization experiment was initiated. Many proteins/complexes undergo chemical and structural changes over time (due to, for example, enzymes or oxidation), so the protein that condenses to form crystals early after drop setup (or using freshly purified protein) may differ from the protein that forms crystals long after setup (or using ‘old’ protein). The concentrations of protein-degradation products and aggregates within a drop increase over time. These can strongly modify nucleation and growth (Caylor *et al.*, 1999 ▸), so that the crystal shapes/morphologies that appear early in a crystallization experiment often differ from those appearing much later.

### 3.4. Crystal slippage during storage and data collection

Without frozen solvent to fix them in place, crystals at room temperature will slip and settle relative to the sample support between mounting and the end of data collection. The rate of slippage depends on the crystal size, the viscosity of the surrounding liquid, the separation between the crystal and the sample support and the area of near-contact between the crystal and the support. Slippage during data collection can affect which portions of a crystal are irradiated and for how long, especially when the X-ray beam is small and especially when the crystal is also small and may move out of the beam. This affects recorded Bragg peak intensities directly through changes in diffracting volume and indirectly through changes in the spatial distribution of radiation damage within the crystal. Slippage may also affect the orientation of a crystal relative to the sample support and beam; because room-temperature crystal mosaicities tend to be very small (<0.02°), even small slip-related orientation changes can have a large effect on the intensities measured in each frame. Mounting crystals in high-viscosity oils (for example Cargille NVH immersion oil or Paratone oil), being careful to minimize excess oil, mounting crystals nearly dry (in an atmosphere that maintains their hydration) with as little liquid as possible between the crystal and the support, mounting crystals on flat films rather than within open loops or on curved (for example capillary) surfaces and using smaller crystals can all help to reduce motion to acceptable levels.

### 3.5. Interpreting weak electron-density features

While room-temperature electron-density maps may show substantial differences from those acquired at cryogenic temperatures, interpreting these differences may not be straightforward. Aside from general increases in atomic *B* factors, room-temperature maps may show different dominant main-chain conformations (for example of mobile loops and flaps), different dominant side-chain conformations and additional, weaker electron density suggesting alternative lower-occupancy main-chain and side-chain conformations (Fraser *et al.*, 2009 ▸; Keedy *et al.*, 2015 ▸, 2018 ▸). Near active sites, additional conformational heterogeneity at room temperature may reveal minority conformations important in ligand binding, and conformational heterogeneity remote from the active site may provide insight into allosteric binding and communication within the molecule (van den Bedem *et al.*, 2013 ▸; van Zundert *et al.*, 2018 ▸).

Comparison of the effects of cryocooling and dehydration shows that in both cases contraction of the unit cell and the resulting changes in molecular packing can account for a substantial fraction of observed conformational remodeling (Atakisi *et al.*, 2018 ▸). In response to either perturbation, the unit-cell volume usually contracts by much more than the protein volume, so packing interactions are increased, room for fluctuations into alternative conformations is reduced and previously dominant conformations may become sterically hindered (Juers & Matthews, 2001 ▸, 2004*a* ▸,*b* ▸). While increased packing may have the greatest effects on surface regions of intermolecular contact, effects may also be seen in remote surface regions (for example when two interior surface regions move into closer proximity) and in interior regions, communicated by force and interaction chains that exist within the molecule (Fraser *et al.*, 2011 ▸; van den Bedem *et al.*, 2013 ▸). Room-temperature maps thus may contain more biologically relevant conformational information, but the observed conformations may still be strongly modulated by packing inter­actions in ways that may not be obvious from visual inspection.

All of these effects should be less pronounced in crystals with higher solvent contents, in crystals with larger solvent cavities and when the regions of primary interest (for example the active site) are remote from regions of intermolecular and intramolecular contact. Collecting and comparing data from multiple crystal forms/molecular packings can help distinguish intrinsic and packing-modulated conformations (Phillips, 1990 ▸; Longhi *et al.*, 1996 ▸; Kondrashov *et al.*, 2008 ▸). When only a single crystal form is available, data from large numbers of crystals of a given crystallographic form can be binned by unit-cell dimensions and the resulting models compared to identify conformational heterogeneity that is sensitive to packing.

### 3.6. Maximizing diffraction signal to background ratio

Reported diffraction resolutions at room temperature have almost always been worse, and often much worse, than those for otherwise similarly prepared crystals at cryogenic temperature. This makes it harder to robustly observe weak electron-density features associated with thermally populated alternative conformations that may be important in enzymatic mechanism or allosteric communication. Some loss of Bragg diffraction intensity at higher resolution is expected due to increased thermal disorder, as reflected in increases in Wilson *B* factors and in diffuse scatter. However, much of the resolution loss at room temperature has been associated with non-optimal sample support and data-collection setups.

Protecting crystals from dehydration has typically involved glass or polymer sealing tubes/films and/or oil. These cause much larger background scatter than is achievable using cryocooled crystals mounted on thin microfabricated polymer films or on nylon loops when excess surface liquid is removed prior to cooling.

Larger degradation of recorded Bragg to background diffraction intensity ratios can arise from the finite source sizes and X-ray beam divergences of both synchrotron and especially laboratory sources (Nave, 1999 ▸). For a crystal illuminated using a monochromatic, nondivergent (parallel) X-ray beam and rotated about an axis φ perpendicular to the beam, strong diffraction in each Bragg reflection will be observed over a range of φ angles Δφ_*hkl*_ determined by the crystal mosaicity and by the Lorentz factor for each reflection (which is between 1 and ∼2 for high-resolution reflections away from the plane defined by the incident beam and rotation axis). Each detector frame records crystal diffraction for a range of angles Δφ. Assuming for simplicity that the background intensity is independent of φ, the ratio of recorded (integrated) Bragg:background intensity will be maximized by recording frames with Δφ ≤ Δφ_*hkl*_; for larger oscillation angles per frame, the Bragg to background ratio will be ∝ 1/Δφ and will decrease with increasing oscillation angle per frame. This is a key motivation behind data collection using fine φ-slicing (Mueller *et al.*, 2012 ▸).

However, the X-ray beams produced by typical laboratory sources have large horizontal and vertical divergences. The X-ray beams produced by the synchrotron source beamlines used for most of the cryocrystallography era have had modest vertical divergences (∼0.02°) but large horizontal divergences (∼0.2°), reflecting the large ratio of horizontal to vertical source size typical of these sources. For a crystal with a mosaicity smaller than the beam divergences, strong Bragg reflections will be observed over a range of angles determined by the divergence ΔΨ (specifically, a diffraction direction-dependent combination of vertical and horizontal divergences, dominated by the latter except near the plane perpendicular to the plane of the incident beam and rotation axis). The maximum ratio of Bragg to background intensity will then be recorded by setting Δφ ≃ ΔΨ, and this ratio will be reduced from the parallel-beam case by a factor Δφ_*hkl*_/ΔΨ, the ratio of the intrinsic reflection oscillation width to the divergence. This also holds true when stills rather than oscillations are recorded.

Crystals at cryogenic temperatures typically have mosaicities of ∼0.2° or larger (Svensson *et al.*, 2019 ▸), so that at synchrotrons it has been mosaicity rather than divergence that usually limits the achievable Bragg to background diffraction ratio. However, well-ordered crystals at room temperature have mosaicities of ∼0.02° and sometimes ∼0.002°, which are comparable to the intrinsic angular spread generated by silicon monochromator crystals (Shaikevitch & Kam, 1981 ▸; Dobrianov *et al.*, 1999 ▸). In this case, beam divergence has been limiting (Supplementary Fig. S9).

To appreciate the impact of this, suppose that a crystal has Wilson *B* factors of 15 and 25 Å^2^ and mosaicities of 0.2° and 0.02° at 100 K and room temperature, respectively, and that diffraction data are recorded by fine φ-slicing in each case. The increase in *B* factor reduces the room-temperature structure factors at 1.5 Å by a factor of ∼3. If the oscillation width Δφ is set by a beam divergence of ∼0.2° at both temperatures, then the recorded Bragg to background intensity ratio will also be smaller by a factor of ∼3. However, if the beam divergence is 0.02° and the step size is set to match the mosaicity at each temperature, then the angular range over which background is recorded at 1.5 Å can be reduced by a factor of ∼10 at room temperature, offsetting the decrease in integrated Bragg peak intensity and increase in thermal diffuse scatter, and allowing more accurate measurement of weaker reflections.

## 4. Advances addressing challenges in room-temperature crystallography

### 4.1. X-ray sources and detectors

Advances in synchrotron X-ray sources, beamline optics and hardware, and X-ray detectors allow useful data to be obtained from more crystals, more and higher quality data to be obtained from each crystal, and more data to be obtained per unit beamtime. These advances are particularly relevant to room-temperature crystallography.

The combination of storage-ring beam currents of a few hundred milliamps and undulator insertion devices has been delivering total monochromatic (Δ*E*/*E* < 10^−3^) photon fluxes of ∼10^12^–10^13^ photons s^−1^ for nearly two decades. Microfocusing X-ray optics have delivered beams ranging from a few micrometres to a few tens of micrometres in diameter, with typical fluxes of 10^11^–10^12^ photons s^−1^, for a comparable time. Corresponding dose rates of ∼0.1 to >10 MGy s^−1^ allow data collection to the room-temperature dose limit in ∼1 s or less. Small beams allow the matching of the beam size to the size of small crystals, reducing background scatter from the surrounding liquid, sample support and air (Moukhametzianov *et al.*, 2008 ▸; Evans *et al.*, 2011 ▸). Small beams also allow separate interrogation of different parts of a crystal (which may be bent, cracked, twinned, part of a cluster *etc.*; Dobrianov *et al.*, 1999 ▸) and data collection from only the most well ordered regions (Bowler *et al.*, 2010 ▸, 2015 ▸).

Advances in single-photon-counting detector frame rates (∼10^3^ Hz) and read-out times together with advances in goniostats/sample-rotation stages have enabled fast data collection via fine φ-slicing. Instead of collecting data for, for example, 90° of sample rotation φ using 90 frames with 1° of rotation per frame, the rotation per frame is chosen to be 1/3 to 1/2 of the oscillation width of higher resolution reflections (Mueller *et al.*, 2012 ▸) which is set by the crystal mosaicity and the beam divergence and is generally much less than 1°, with a corresponding increase in the number of frames per data set and reduction in integrated background beneath Bragg reflections in each frame. At room temperature, data collection from a single crystal to its dose limit can be completed in seconds or less, so crystal slippage during data collection is negligible. Significant slippage may still occur when crystals are out of the beam, for example during long raster or helical scans or between crystal centering and data collection.

The dramatically more brilliant beams that can be delivered by newer, lower emittance sources such as NSLS-II, MAX-IV and the recently commissioned ESRF-EBS, and that will be provided by APS-U, are ideally suited to optimizing room-temperature data collection. For example, the undulator source for the FMX beamline at NSLS-II (Schneider *et al.*, 2021 ▸) has an ∼40 µm (horizontal) × 4 µm (vertical) source size and divergences of 0.021 mrad (h) and 0.009 mrad (v). The small vertical and horizontal source size and divergence deliver unfocused 10 µm diameter X-ray beams at the sample position with ∼3.5 × 10^12^ photons s^−1^ and divergences of 0.5 mrad (h) × 0.5 mrad (v) [or 0.03° (h) × 0.03° (v)]. The same photon flux can be focused to a beam of size 1.5 µm (h) × 1 µm (v) with a larger divergence of 3 mrad (h) × 2 mrad (v) [or 0.17° (h) × 0.11° (v), which are non-optimal for crystals at room temperature]. The corresponding average dose rates at 10 keV delivered to typical protein crystals within the FWHM of the beam are then ∼12 and ∼820 MGy s^−1^ (Supporting Information Section S2), allowing data collection to the ∼200 kGy room-temperature dose limit (Section 3.1) in ∼16 and ∼0.2 ms, respectively. In these short data-collection times, some of the ‘excess’ radiation damage associated with free-radical diffusion and structural relaxations of protein and packing that occur at room temperature can be outrun (Warkentin *et al.*, 2013 ▸, 2017 ▸; de la Mora *et al.*, 2020 ▸), reducing the ‘room-temperature penalty’. Some additional reduction in damage per dose may result when using micrometre and smaller beams due to photoelectron escape from the illuminated sample volume (Nave & Hill, 2005 ▸; Sanishvili *et al.*, 2011 ▸; Finfrock *et al.*, 2013 ▸).

Beamline ID29-EBSL8 at ESRF is now delivering ‘pink’ (Δ*E*/*E* ≃ 3%) photon fluxes of ∼10^15^ photons s^−1^ in ∼400 × 600 nm beams gated by a fast chopper (down to 10 ms), with diffraction recorded using a JUNGFRAU 4M detector framing at 1.1 kHz. Corresponding dose rates at 10 keV of ∼1.6 TGy s^−1^ allow data collection to a dose of ∼200 kGy (the room-temperature dose limit measured using dose rates below ∼1 MGy s^−1^) in ∼0.1 µs. The maximum tolerable dose at room temperature when data are collected on timescales of a few milliseconds increases by a factor of 1.5–2 relative to the low-dose-rate/long-timescale limit (Warkentin *et al.*, 2013 ▸, 2017 ▸; de la Mora *et al.*, 2020 ▸). Even if the timescales for the diffusion and relaxation processes that increase room-temperature radiation-sensitivity have a very broad distribution (Warkentin *et al.*, 2011 ▸), decreasing the data-collection timescale by an additional four orders of magnitude should increase the maximum tolerable doses over those measured at dose rates below 1 MGy s^−1^, perhaps by as much as a factor of 10 (Supplementary Fig. S7).

X-ray free-electron laser (XFEL) sources provide another approach to addressing radiation damage in room-temperature data collection (Suga *et al.*, 2015 ▸; Spence, 2017 ▸; Stauch & Cherezov, 2018 ▸; Nass, 2019 ▸; Barends *et al.*, 2022 ▸). Current sources generate highly brilliant X-ray pulses of ∼5–50 fs, each containing ∼10^11^–10^12^ photons in focused spot sizes of down to ∼1 µm in diameter (Gati *et al.*, 2017 ▸; Sierra *et al.*, 2019 ▸; Keable *et al.*, 2021 ▸; Barends *et al.*, 2022 ▸). Doses per pulse (averaged over the beam FWHM) at 10 keV on crystallo­graphy stations are ∼30 MGy (for example for the MFX station at the LCLS, with 1 × 10^12^ photons per pulse and a 3.7 µm beam diameter) and as large as ∼220 MGy s^−1^ (for example for the microfocusing CXI station at the LCLS, with 1 × 10^12^ photons per pulse and a 1.3 µm beam). A single pulse can vaporize an ∼20 µm liquid jet (Stan *et al.*, 2016 ▸) and in a large crystal disrupts an ∼30 µm diameter region around it (Hirata *et al.*, 2014 ▸; Suga *et al.*, 2015 ▸). However, with such a short pulse, X-ray interaction with a crystal is complete before the atoms and their electron clouds can respond to the energy deposited by inelastic processes, so that diffraction from that single pulse captures the crystal in a state that is not only free of ‘global’ damage but is also largely free of site-specific changes (for example reduction of metal centers; Lomb *et al.*, 2011 ▸; Barty *et al.*, 2012 ▸; Suga *et al.*, 2015 ▸). For crystals comparable to or smaller than the beam size, current XFEL crystallography stations allow diffraction of ∼2 and 10^2^ times (and up to 20 and 10^3^ times) more photons per unit sample volume at cryogenic and room temperature, respectively, than is feasible given radiation-damage limits when using synchrotron source beams of comparable size delivering dose rates below ∼10 MGy s^−1^. This eliminates radiation damage as a rationale for cryogenic temperature data collection.

However, radiation-damage-free XFEL data often come at a considerable cost. Each pulse generates a single frame of diffraction from the crystal region within its footprint (perhaps 3 µm in diameter) but disrupts any crystal or solution within a ∼30 µm diameter (Hirata *et al.*, 2014 ▸) or within a volume ∼100 times larger than the illuminated volume. The volume disrupted is determined by the pulse energy, which is proportional to the number of photons per pulse. Concentrating these photons into a smaller diameter beam increases the dose and diffracted photons per unit sample volume, but also increases the ratio of disrupted to illuminated sample by the same factor. To make an ideally efficient use of crystals, each crystal should have a size equal to the beam size, be separated from other crystals by the ‘blast diameter’ and be mounted ‘dry’ on an X-ray transparent support. As discussed in Section 4.2, these conditions are not achieved in current approaches for sample delivery into XFEL beams, and generally <1% (and often ≪1%) of the available volume of crystals is illuminated. Furthermore, since the crystal orientation is random and unknown, ensuring adequate coverage of reciprocal space and dealing with reflection partiality typically involves the collection of 10^4^–10^5^ frames from an equal number of crystals, even when crystals of 10 µm or larger may be available.

Consequently, at room temperature current XFEL stations allow ∼10^2^ more photons to be scattered per unit illuminated crystal volume, but only 10^−2^ or less of the total available volume of crystals may be illuminated in most experiments. Unless the crystals are small and extremely abundant, or the goal is to eliminate site-specific radiation damage, current- and next-generation high-brilliance synchrotron sources are likely to be superior for ‘static’ room-temperature crystallography.

### 4.2. Sample delivery for data collection

As discussed by Fischer (2021 ▸) and in several excellent reviews of serial crystallography (Grünbein & Kovacs, 2019 ▸; Zhao *et al.*, 2019 ▸; Martiel *et al.*, 2019 ▸; Cheng, 2020 ▸), many sample-mounting and sample-delivery systems suitable for room-temperature crystallography, of widely varying complexity, have been developed in the last 20 years. Table 2 ▸ gives a summary of methods, some of which are illustrated in Supplementary Figs. S1–S6.

For measurement of one or a few crystals, the glass capillary method (Supplementary Fig. S1) that was dominant through the early 2000s is obsolete except when absolute impermeability to gas (for example for oxygen-sensitive crystals) is required. Crystals can instead be harvested onto a nylon or microfabricated loop as in cryocrystallography, excess liquid blotted or wicked away to prevent slippage and then covered by an optically and X-ray transparent PET or Kapton tube that contains reservoir solution at one end and that seals at the other end to a modified goniometer base to prevent dehydration (Kalinin *et al.*, 2005 ▸). Crystals within the tube remain hydrated for weeks (but not months) and can be mounted and shipped to the synchrotron (with the caveats noted by Fischer), and crystal centering is as straightforward as in cryocrystallography. X-ray data collected by shooting through the ∼25 µm wall tubing has substantially less background scatter than with glass X-ray capillaries, but background may become an issue for crystals of a few micrometres in size.

For the delivery of 10^4^–10^7^ ‘microcrystals’ (*i.e.* where the crystal dimensions may range from ∼30 µm to ∼200 nm) into an X-ray beam, liquid jets (Supplementary Fig. S2; see, for example, Pandey *et al.*, 2021 ▸) are used at XFEL sources. LCP/high-velocity extrusion (HVE) injectors (Supplementary Fig. S3; see, for example, Weierstall *et al.*, 2014 ▸) and ‘drop-on-tape’ systems (Supplementary Fig. S4; see, for example, Butryn *et al.*, 2021 ▸), in which crystal-containing drops are deposited onto a moving X-ray transparent ‘tape’ that carries them into the X-ray beam, are used at both XFEL and synchrotron sources. Both sets of approaches require complex apparatus at the beamline and multiple staff on site for data collection. Because of the low XFEL pulse repetition rate (when operating at pulse energies/repetition rates that maximize diffracted photons per crystal), only a very small fraction, perhaps 0.02% or less, of a liquid-jet volume ever sees X-rays, and the use of crystals is very inefficient. LCP/HVE injectors and drop-on-tape systems are both more efficient. However, injected stream diameters (∼30 µm) or drop diameters (∼250 µm) (Butryn *et al.*, 2021 ▸) are much larger than typical X-ray beam sizes at XFELs (∼1–3 µm) or synchrotrons (∼10 µm). In LCP/HVE and drop-on-tape experiments at XFELs, less than ∼1% of the stream volume and ∼0.1–0.01% of the drop volume, respectively, sees X-rays, and multiple crystals may be in the beam path if the crystals are not sufficiently diluted. All three methods can only present each crystal to the beam in a single (unknown) orientation. All place more liquid/gel in the beam path and generate substantially more background scatter than is achievable using the fixed-target approaches discussed below. After crystallization hits suitable for structural study using conventional loop-based methods have been obtained, generating adequate numbers of suitably small crystals typically requires significant additional optimization and filtering (using metal screens) of crystal-containing solutions to remove crystals that are too large.

As the growing number of structure depositions generated using these sample-delivery methods attests, the challenges posed by inefficient protein and crystal use are usually not insurmountable and can be irrelevant for previously well studied model systems. At the same time, the rapidly expanding use of single-particle cryo-EM and the emergence of micro-electron diffraction (micro-ED), both of which require orders of magnitude less biomolecule sample, suggests that these sample-delivery methods for crystallography will not be a first or even a second choice for most structural biologists.

What if the goal is to determine high-resolution room-temperature structures with as little total effort in protein production, crystallization, optimization, soaking and handling as possible, and with as parsimonious and as complete use (to ensure identification and measurement of all polymorphs present; Ebrahim *et al.*, 2019 ▸) of available crystals as possible? Most crystallization will continue to be performed using 24-, 72-, 96-, 384- or 1536-well microplates or using LCP sandwich plates. A reasonable goal is then to get every crystal in every drop into the X-ray beam and to maximize the amount and quality of data that can be collected from each crystal.

A variety of ‘fixed-target’ approaches for the delivery of ∼10^2^–10^5^ plate-grown crystals into an X-ray beam at room temperature have been developed that achieve these goals to varying extents. Some use large (>10 cm^2^) arrays of cells that are loaded on site and then held in the beam using custom apparatus (Mueller *et al.*, 2015 ▸; Owen *et al.*, 2017 ▸; Doak *et al.*, 2018 ▸; Wierman *et al.*, 2019 ▸; Mehrabi *et al.*, 2020 ▸).

Alternative fixed-target approaches attempt to make the room-temperature sample-preparation and data-collection experience for end users similar to those in cryocrystallography and to exploit the existing cryocrystallography infrastructure (Roedig *et al.*, 2015 ▸, 2016 ▸; Baxter *et al.*, 2016 ▸; Guo *et al.*, 2018 ▸; Lieske *et al.*, 2019 ▸; Karpik *et al.*, 2020 ▸; Illava *et al.*, 2021 ▸). These use polymer or silicon supports held in cryocrystallography-compatible goniometer bases, and in some designs the support dimensions are compatible with the grippers of beamline sample automounters. Example supports (Supplementary Figs. S5 and S6) include ∼100 µm thick polycarbonate sheet with circular holes (Baxter *et al.*, 2016 ▸), silicon ‘chips’ with 10–20 µm thick silicon membranes having an array of through-holes (Roedig *et al.*, 2015 ▸, 2016 ▸; Lieske *et al.*, 2019 ▸) and thin (4–20 µm) polyimide or cylic olefin copolymer films micropatterned with holes, wells and other features and held in a rigid frame (Guo *et al.*, 2018 ▸; Karpik *et al.*, 2020 ▸; Illava *et al.*, 2021 ▸). For supports with suitable areas (several mm^2^), an entire crystallization drop can be transferred onto the support (Lieske *et al.*, 2019 ▸; Karpik *et al.*, 2020 ▸; Illava *et al.*, 2021 ▸) using pipettes, syringes or microtools with metal or flexible polymer film tips, with little risk of crystal loss. Excess liquid can be drawn away through holes in the sample support by backside blotting or suction (Mueller *et al.*, 2015 ▸; Roedig *et al.*, 2016 ▸; Karpik *et al.*, 2020 ▸; Illava *et al.*, 2021 ▸), minimizing X-ray background and crystal slippage. The support films can be patterned with features to help to localize crystals in particular regions, to guide them towards the holes or to help keep them randomly distributed; experiments show that these features need only be a few micrometres high to be effective for crystals of up to ∼20 µm thick (Illava *et al.*, 2021 ▸). The entire sample-support film then remains thin and retains excellent optical and X-ray transparency. Data can be collected from the entire volume of every crystal on the support and over a wide oscillation range (>90°) so that preferential orientation on the support is only a minor issue.

Fixed-target supports are typically sealed using ∼5 µm clear films (typically of Mylar) to minimize evaporation during data collection while producing a modest and unstructured X-ray background. Water-vapor transmission rates of ∼0.1–1 µl per day at ambient humidity and the small residual liquid volume on the supports can result in appreciable crystal dehydration in tens of minutes to hours. Additional, thicker films of more impermeable polymers that are opaque and/or highly oriented or crystalline (and thus unsuitable for X-ray data collection) such as PTFE, PVDC and polypropylene can be applied on top of the Mylar film for storage and shipping. For longer term storage and shipping, crystals on supports can be stored in devices that contain a substantial volume (∼1 ml per sample) of solution (for example reservoir solution) to match the water activity in the crystals or at least to minimize the r.h. difference between the crystals and ambient air that drives evaporation. These storage devices can double as *in situ* crystallization devices (Baxter *et al.*, 2016 ▸; Lieske *et al.*, 2019 ▸).

*In situ* crystallization plates, which allow X-ray examination of crystals, provide another approach suitable for crystal diffraction screening but not for slip-free low-background oscillation data collection to the highest possible resolution. Crystallization can be performed directly in/on X-ray sample supports (including fixed-target serial crystallography supports). This is typically attempted once crystallization conditions have been identified by other means (for example screening in 96-well plates), and additional on-support optimization may be required to obtain suitable crystals. A hybrid approach developed by ESRF uses crystallization plates with 12.5 µm films supporting crystallization drops, a laser to cut out the film supporting each drop and a robot to transfer the drop-holding film into the X-ray beam (Cipriani *et al.*, 2012 ▸).

Note that many serial crystallography sample-delivery systems implicitly or explicitly assume that the crystals are small, abundant and of similar size, and that diffraction data will be collected either without oscillation or over only a small angular range. However, when conditions that yield well ordered crystals are identified, the crystals are usually not small; plates and rods with large areas per unit volume are common and data collection over a substantial oscillation range is feasible and desirable. An analysis of over 56 000 crystals examined on the automated MASSIF-1 beamline at ESRF (Svensson *et al.*, 2019 ▸), which has automated (and user-selectable) beam sizes down to 10 µm (Svensson *et al.*, 2018 ▸), indicated an average crystal volume of ∼2 nl and that less than 1% of crystals had volumes of less than 10 pl. These volumes correspond to cubes of sides 132 and 22 µm, respectively. Although obviously biased by the crystals that users chose to send for measurement and by the minimum detectable crystal sizes (likely ∼5 µm), these results give a sense of the spread of crystal sizes that are encountered in routine practice.

As discussed below, to take better advantage of the available pulses [which may come singly at 60–120 Hz or (at the European XFEL) in bunches of hundreds of pulses repeating at 10 Hz], crystals may be delivered in liquid jets, in lipidic cubic phase (LCP) or gel-based high-velocity extrusion (HVE) streams, or in a series of drops on a rapidly moving tape. In this case, each pulse generates a single frame of data corresponding to a single, unknown orientation of each crystal that is ‘hit’. No data can be collected from any portion of the crystal stream or drop that is not illuminated by a pulse.

### 4.3. Minimizing background scatter and maximizing crystal isomorphism

Background X-ray scatter is managed by minimizing all noncrystal sources of scatter along the path of the beam to the detector. Polymers and water/buffer produce similar amounts of scatter per unit length, while air produces ∼10^−3^ less scatter. For polymer or silicon sample supports sealed with polymer films, mechanical strength and vapor impermeability require total polymer thicknesses along the beam path of ∼10–15 µm. Liquid on the support after loading can easily be several times this thickness and should be removed. Air gaps between upstream and downstream vacuum flight tubes of a few centimetres, typical of cryocrystallography beamlines, generate scatter equivalent to 20–30 µm of water or polymer, and can be reduced to ∼6–8 mm while leaving ample room for sample-support oscillations.

Crystal non-isomorphism can be minimized by ensuring consistent crystal hydration, ideally from crystal harvesting through storage and shipping to the end of data collection. Increasing the ambient r.h. during harvesting and mounting reduces the rate of evaporation, but since water activities in as-grown protein crystals correspond to 97% to >99% r.h. (Wheeler *et al.*, 2012 ▸), crystal-size dependent dehydration can still be substantial even in nominally stagnant, well-humidified air. Removing excess liquid to minimize slippage and background scatter increases the rate of crystal dehydration, especially if suction is also employed in liquid removal. Building on earlier workstation designs, particularly by the MPI/Toronto group (Mehrabi *et al.*, 2020 ▸), we have demonstrated a humidified workstation that generates, maintains and allows accurate measurement (to ±0.5%) of near-saturating (97–100%) humidities, that is equipped with a vacuum end with foot-pedal-controlled suction for liquid removal within the humidified enclosure, and that has defogging heaters, external epi-illumination and transmitted light illumination, and a microscope for observation of crystallization trays and the sample support (Illava *et al.*, 2021 ▸).

At the beamline, dehydration can be minimized and support-to-support crystal isomorphism maximized by bathing the sample support in high-humidity gas (Sanchez-Weatherby *et al.*, 2009 ▸; Baba *et al.*, 2013 ▸, 2019 ▸; Bowler *et al.*, 2015 ▸). This is particularly important if data are to be collected over the entire area of the sample support and if oscillations are to be performed at some or all locations; data collection may then take ∼30 min or more, depending upon the sample translation and rotation hardware. Any high-humidity (>90% r.h.) gas stream (Farley *et al.*, 2014 ▸) or enclosure will dramatically extend the working time. For beamlines equipped with a humidified gas stream that can maintain and measure near-saturating humidities at the sample position with an accuracy of ∼1% r.h. or better and with a reproducibility of ∼1% r.h. or better between samples (a significant challenge, since above 95% r.h. most humidity sensors have poor accuracy and show hysteresis), sealing films on the support can be removed and the sample r.h. controlled using the gas stream, reducing background scatter. Humidified gas streams can also restore the hydration and order of mildly dehydrated samples.

### 4.4. Crystallographic analysis and modeling

Crystallographers, especially those studying viruses (see, for example, Grimes *et al.*, 1998 ▸), have long been collecting and merging data from tens to thousands of crystals to obtain complete data sets suitable for structure determination. The challenges of serial crystallography using XFELs have helped to drive the development of robust and easy-to-use software pipelines for handling ∼10^7^ frames acquired from ∼10^5^ crystals in unknown orientations, and for selecting, indexing, scaling and merging subsets into complete data sets suitable for model building and refinement. For *de novo* structure determination, the use of heavy-atom- or selenomethionine-containing crystals for phase determination increases the cost of room-temperature versus cryo-temperature data collection. However, initial phases may now be obtainable using structures predicted using *AlphaFold* (Jumper & Hassabis, 2022 ▸) and *RoseTTAFold* (Baek *et al.*, 2021 ▸) or from lower resolution structures determined using single-particle cryo-EM.

To interpret the additional features associated with alternative conformations present in room-temperature electron-density maps, tools including *Ringer* (which identifies statistically significant density peaks at rotameric positions of side chains; Lang *et al.*, 2010 ▸, 2014 ▸), *QFit* (which autobuilds a parsimonious set of side-chain and main-chain conformations; van den Bedem *et al.*, 2009 ▸; van Zundert *et al.*, 2018 ▸), *Contact* (which identifies networks of side-chain rotamer interactions; van den Bedem *et al.*, 2013 ▸) and full ensemble refinement in *Phenix* (Burnley *et al.*, 2012 ▸) are available, although the final models typically require substantial manual refinement.

### 4.5. Interpreting room-temperature electron density: multi-temperature data collection

Both increased thermal energy (affecting the whole protein and solvent) and reduced packing interactions (at sites of intermolecular and intramolecular contact) lead to increased conformational heterogeneity at room temperature relative to cryogenic temperature. However, accessible conformations, both of surface residues directly involved in intermolecular or intramolecular contacts and of regions remote from those contacts, are often still influenced by crystal packing. The structure visible in room-temperature electron-density maps may then reflect both biologically relevant minority conformations as well as conformations associated with packing.

A powerful approach to distinguishing between the possibilities, especially for residues that are not directly involved in contacts, is to collect data at multiple temperatures (Keedy *et al.*, 2015 ▸, 2018 ▸; Atakisi *et al.*, 2018 ▸; Keedy, 2019 ▸). Structural data sets collected at temperatures above the protein–solvent glass transition near *T* = 200 K facilitate discrimination between ‘static’, temperature-insensitive conformational disorder (for example associated with frustrated interactions), crystal packing-associated changes (which produce changes in the energy landscape that can modify majority conformations) and ‘purely’ temperature-driven changes (which may produce changes in relative occupancy within a fixed energy landscape as well as changes in the landscape arising from, for example, temperature-dependent p*K*_a_ values.) This additional information can help to identify features salient to biological mechanism.

Fortunately, collecting these additional data should be feasible using crystals prepared in the same way and loaded onto the same fixed-target supports as for room-temperature data collection (Warkentin & Thorne, 2010*b* ▸), at least for those fixed-target supports that are compatible with standard high-throughput cryocrystallography beamlines (Coulibaly *et al.*, 2007 ▸; Gati *et al.*, 2014 ▸; Baxter *et al.*, 2016 ▸; Roedig *et al.*, 2016 ▸; Meents *et al.*, 2016 ▸; Guo *et al.*, 2018 ▸; Karpik *et al.*, 2020 ▸; Illava *et al.*, 2021 ▸). At the low-temperature end, conformational evolution and changes in overall and atomic *B* factors are largely complete by 200 K (Doster, 2010 ▸; Keedy *et al.*, 2015 ▸), with only small additional changes occurring on cooling to 100 K. Ice formation inside protein crystals is strongly suppressed during cooling (Moreau *et al.*, 2019*a* ▸) and warming (Weik *et al.*, 2001 ▸) by solvent nanoconfinement within solvent channels. In typical as-grown crystals with no added cryoprotectants, ice is unlikely to form on timescales of ≤10 s even at *T* = 240 K, provided that the external solvent is removed and replaced with oil. At the high-temperature end, proteins eventually unfold and/or degrade. Crystal packing strongly inhibits this, and the structure may remain stable for ≥10 s at temperatures of 330–360 K and possibly higher, provided that the crystal is protected against dehydration by oil and/or by an appropriately humidified, preferably oxygen-free environment (Doukov *et al.*, 2020 ▸).

Cooling and warming of crystals sealed in fixed-target serial crystallography supports can be performed using standard temperature-controlled nitrogen-gas streams available at cryocrystallography beamlines and can be abruptly initiated using an air blade or mechanical shutter or by mechanical translation of the gas-stream source. Diffraction data can then be collected once the sample cools or warms to within a few degrees of the gas-stream temperature, which typically requires <1 s, and data collection can be continued until the diffraction of the crystal degrades either due to radiation damage or to temperature-related processes (Moreau *et al.*, 2019*a* ▸; Doukov *et al.*, 2020 ▸). With typical maximum synchrotron beamline flux densities, data collection to the dose limit within the beam footprint can be completed in ≪1 s, and oscillation or helical scan data can be collected from a 30–50 µm crystal in seconds. For the model proteins studied to date the half-dose *D*_1/2_ decreases by roughly a factor of two for each 30 K increase in temperature above 200 K, so the radiation-damage penalty for data collection above room temperature is modest. Multi-temperature data collection should also be feasible when samples are delivered using LCP/HVE injectors and drop-on-tape systems, but will require high cryoprotectant concentrations (which may perturb the conformations of interest) to prevent ice formation in the surrounding liquid/gel at lower temperatures and humidity-controlled gas streams to prevent dehydration at higher temperatures.

Consequently, collecting diffraction data from ‘native’, cryoprotectant-free crystals at temperatures between ∼220 and ∼350 K should generally be feasible, and collecting data between 250 and 330 K should be routine, which is a wider temperature range than is typically feasible using other all-atom structural probes.

## 5. Room-temperature crystallography: from last resort to a workhorse tool in structural biology?

As we have seen, room-temperature data collection overcomes many issues in current cryocrystallographic practice at modest cost. Room-temperature structure determination does require a larger total crystal volume, but even so one or a few crystals are often sufficient. No crystals and no effort need to be expended in identifying suitable cryoprotection and cryocooling conditions, and crystal screening and optimization can be expanded to include more cryoprotectant-free conditions. Advances in sample supports and sample-loading tools/workstations make it feasible to get every crystal in every drop into the X-ray beam with little or no dehydration-induced non-isomorphism and minimal background scatter. Advances in X-ray sources, in beamline optics and detectors, and in analysis tools for handling sparse, weakly exposed frames (Lan *et al.*, 2017 ▸) make it feasible to obtain useful diffraction from ordered crystals of sizes of ∼1 µm and larger (Gati *et al.*, 2017 ▸). Beams with diameters comparable to or smaller than the crystal sizes (down to ∼1 µm) and with both horizontal and vertical divergences smaller than the room-temperature crystal mosaicities, combined with fine φ-slicing in steps of ∼1/3 to 1/2 the (very small) mosaicity, all help to maximize recorded diffraction signal to background and give resolutions at room temperature that should approach those obtained at cryogenic temperature. Sample-storage boxes with liquid reservoirs and standard commercial temperature-controlled packaging allow the shipping of samples mounted in the home laboratory to the synchrotron for measurement. The gas streams available at many beamlines eliminate/manage crystal dehydration and allow data collection at multiple temperatures.

Furthermore, crystals at room temperature always exhibit less static disorder than cryocooled crystals, their diffraction patterns are never contaminated by ice and their internal solvent spaces can be cryoprotectant-free and thus have the potential to deliver better data quality. Crystals at room temperature exhibit more thermal disorder, but this disorder contains information about conformational heterogeneity, some of which may be important in function. Collecting data from multiple crystal polytypes, including weakly packed, high-solvent-content forms that are more likely to be damaged by cryoprotection and cooling, can be more straightforward at room temperature, allowing a comparison of conformations to identify those of the greatest biological relevance. Finally, advances in crystallographic analysis and modeling software have made processing diffraction frames from large numbers of crystals to assemble high-quality data sets and mining these data sets for information about room-temperature conformational heterogeneity more straightforward. Advances in structure prediction may reduce the number of crystals that are required for *de novo* structure determination.

For all of these reasons, the prejudices and practicalities that have driven the near-exclusive use of cryocooled crystals in crystallography are becoming less valid, and the opportunity costs of not collecting room-temperature data are growing. What remaining obstacles must be overcome before room-temperature crystallography returns to the mainstream of structural biology practice?

Most room-temperature data collection involves the transport of crystallization materials and/or loaded plates to the synchrotron and harvesting and mounting crystals at the beamline. For room-temperature crystallography to achieve its potential, we need to be able to prepare and mount crystals in the home laboratory and ship them to the synchrotron for remote, high-throughput data collection. We need a standardized, mail-in workflow with standardized tools similar to those for cryocrystallography and, ideally, that exploits as much of the existing cryocrystallography infrastructure as possible. We need beamlines that are configured to handle room-temperature samples in a high-throughput way, with X-ray beam and sample-environment properties optimized for those samples.

Home laboratories should have access to humidified workstations, humidified gas streams or other devices (including home-built humidified ‘tents’) to ensure that samples remain fully and uniformly hydrated during harvesting, mounting and sample-support sealing. The critical aspect here is that the humidity should be maintained as close to the water activity inside the crystals as possible, which typically means close to saturating humidity (100% r.h.).

A set of standard sample supports for holding one or multiple crystals for room-temperature data collection should be approved for use at multiple synchrotrons, and these approvals and best practices publicized to their user communities. Good candidate supports, including those developed for fixed-target serial crystallography, exist. Vetting, approval and publicizing this approval must continue.

A set of standard shipping ‘cassettes’/storage boxes and packaging that maintain sample humidity and temperature and that facilitate (ideally, automated) sample transfer and handling at the synchrotron should be vetted and approved for use at multiple synchrotrons. A UniPuck-format storage container that holds sample supports in a plane with the goniometer base down and grippable from above might be most suitable to take advantage of existing high-throughput cryocrystallography hardware. The SSRL-developed sample cassette and serial crystallography *in situ* crystallization/storage box (Baxter *et al.*, 2016 ▸) provide access to the gonio­meter base bottoms, and a mechanism flips and regrips the sample supports for placement on the beamline goniostat.

Synchrotron beamlines should be configured with room-temperature (or temperature-regulated) sample-loading stations that hold approved cassettes/storage boxes in position for automated sample loading, with both room and cryo-temperature sample loading (if feasible) using existing beamline sample-handling robots. High-throughput data collection with minimal operator intervention will require samples sealed by (nominally) vapor-impermeable thin films in the home laboratory prior to shipping. To minimize dehydration via diffusion through sealing films during beamline storage and during long data-collection scans, the sample-loading station should be humidified and the sample placed in a humidified gas stream during data collection. Humidities of >90% and preferably >95% r.h. will provide long working times, and can be generated and (inaccurately) measured using inexpensive devices. To maximize diffraction signal to noise, beamlines should have minimal air gaps at the sample position, minimal horizontal and vertical beam divergence (which may require defocusing at less brilliant sources) and beams comparable to or smaller than the crystal size.

Crystals can be prone to degradation during long-term storage at room temperature. ‘Just-in-time’ nucleation and growth of diffraction-quality crystals for previously scheduled beamtime will often be impractical. Thus, an ultra-rapid-access process for remote-access beamtime, whereby crystals can be shipped whenever and as soon as they are prepared and then measured within approximately two days should be developed.

Finally, to support the growing community of researchers interested in utilizing room-temparature (and variable-temparature) crystallography, the BioSync website should be upgraded to provide a directory of all synchrotron and XFEL beamlines specifically equipped for high-throughput/serial room-temperature crystallography, their sample-support requirements and their relevant capabilities (including X-ray beam parameters).

## Supplementary Material

Details of dose rates and dose estimates at various X-ray sources. DOI: 10.1107/S2059798322011652/rs5006sup1.pdf

## Figures and Tables

**Table 1:** Advantages and challenges of collecting X-ray data from biomolecular crystals at room temperature versus cryogenic temperature

**Advantages of room-temperature data collection**
Closer to native conformational ensembles, intramolecular allosteric communication and ligand-interaction states. Multi-conformer structure refinements are more useful.
No cryoprotectants required. Increased electron-density contrast between protein and solvent increases Bragg diffraction intensity. Clean solvent spaces preserve water structure and reduce misidentification of ligands.
No cryoprotectant soaks required. No osmotic shock.
No cryocooling required and no sample variability due to irreproducible cooling. Much lower mosaicities enable fine φ-slicing and can increase the Bragg peak signal to background recorded in each frame.
No crystal damage by ice formation. No perturbation of recorded Bragg peak intensities by ice diffraction.

**Challenges**
Increased rate of radiation damage with dose and smaller maximum tolerable doses. Data collection from a larger volume of crystal and possibly multiple crystals required.
Crystal damage and nonisomorphism due to dehydration. Must preserve full and constant crystal hydration to the end of data collection.
Mechanical fragility, especially of rod and plate morphologies and high solvent-content crystal forms.
Crystal transport to the X-ray source.
Increased thermal disorder (some functionally relevant), increased thermal contribution to *B* factors, increased diffuse background scatter and reduced Bragg intensity at large scattering angles.
Crystal slippage on fixed-target supports between mounting and the end of data collection.

**Table 2:** Comparison of methods for delivering crystals into the X-ray beam for room-temperature crystallography, based on their current state of development Background-scatter evaluation assumes, for fixed-target approaches, that excess liquid around crystals has been minimized by blotting or suction. For alternative comparisons of these methods, see Table 1 in Cheng (2020 ▸) and Table 1 in Martiel *et al.* (2019 ▸).

Sample-delivery method	No. of crystals	Crystal size range	Crystal targeting	Data-collection modes	Total volume of crystals probed by X-rays (%)	Background scatter	*T* = 100 K and multi-*T* data collection?
Fixed targets							
Glass capillary	1–∼10	<2 mm	Center on each crystal; raster	Full (360°) oscillation; helical scanning	∼100%	High	Maybe
Loop/mesh + polymer capillary	1–∼10^2^	<1 mm	Center on each crystal; raster	Full oscillation; helical scanning	∼100%	Moderate	Yes
Loop/mesh + humidified gas stream	1–∼10^2^	<1 mm	Center on each crystal; raster	Full oscillation; helical scanning	∼100%	Low	Yes
Solid support with array of holes/wells/windows							
Small area/HT MX compatible	1–∼10^4^	<hole size (∼10–20 µm)	Step and repeat	Partial oscillation (∼10–90°)	0.1–10%	Low	Yes
Large area	1–∼10^6^	<hole size (∼10–20 µm)	Step and repeat	Fixed orientation	0.1–10%	Low	No
X-ray transparent support (polymer, SiN)							
Small area/HT MX compatible	1–∼10^4^	<2 mm	Center on each crystal; raster	Nearly full oscillation; helical scanning	∼100%	Low	Yes
Large area	1–∼10^6^	<2 mm	Center on each crystal; raster	Fixed orientation	∼100%	Low	No
Jets, streams, tape							
Liquid jets	>10^9^	<jet diameter (<10 µm)	Jet moves through beam at ∼30 m s^−1^	Fixed orientation	<0.01%	Low	No
LCP and LVE injector streams	>10^6^	<gel diameter (<30 µm)	Gel moves through beam at mm s^−1^	Fixed orientation	<1%	Moderate	No
Drop-on-tape	>10^6^	<drop diameter (<200 µm)	Drops move through beam at mm s^−1^	Fixed orientation	<0.1%	Moderate	Maybe
