# Supplementary material for: Determining biomolecular structures near room temperature using X-ray crystallography: concepts, methods and future optimization
Source: Acta Crystallogr D Struct Biol. 2023 Jan 1;79(Pt 1):78–94. doi: 10.1107/S2059798322011652 (PMC9815097; doi:10.1107/S2059798322011652)
Supplement: Supplementary file 1 [file d-79-00078-sup1.pdf]

Acta Cryst D STRUCTURAL BIOLOGY

Volume 79 (2023)

Supporting information for article:

Determining biomolecular structures near room temperature using X-ray crystallography: concepts, methods and future optimization

Robert E. Thorne

Supporting information

S1. Example approaches for sample delivery in room and near-room temperature X-ray crystallography.

Figures S1-S6 schematically illustrate approaches to delivering crystals into the X-ray beam for room-temperature and near room-temperature crystallography.

S2. Estimation of doses, dose rates, and radiation damage limits in synchrotron and XFEL data collection from protein crystals at room temperature.

Maximum tolerable doses – total energy deposited per unit crystal volume - for crystallographic data collection in general depend on the initial or target resolution, data collection temperature, dose rate, crystal size, the specific protein and crystal form, and also upon the pattern of crystal irradiation. Manifestations of damage include an overall decay in Bragg peak intensities, a more rapid decay of peak intensities at higher (smaller numeric value) resolution, an increase in scaling B factors, an increase in diffuse intensity, an increase in crystal mosaicity, and an increase in unit cell volume. In real space, these manifest as an overall loss of resolution in electron density maps, and site-specific structural changes at, e.g., metal centers and disulfides.

S2.1. Maximum doses for crystallographic data collection at cryogenic and room temperature using synchrotron sources

At cryogenic temperatures, the half-dose $D_{1/2}$ for data collection to a resolution of 1.5-2 Å is roughly 10-15 MGy, as originally measured by Teng and Moffat and as found in many subsequent measurements. This is consistent with the limit originally suggested by Henderson but is roughly 1/3 the "Garman" limit of ~45 MGy, which was incorrectly determined. Available evidence suggests that the half-dose in each resolution shell varies roughly as the square of the resolution, consistent with results in cryo-EM and with simple models for the radiation damage process. The half-dose and maximum tolerable dose at cryogenic temperatures appear to be consistent across proteins and crystal forms, with no major outliers having been rigorously documented. The half dose and maximum tolerable dose at cryogenic temperatures are independent of dose rate up to at least 50 MGy/s, and are roughly independent of temperature below roughly 140 K. Photoelectron escape from the illuminated volume can reduce the rate of damage per dose and increase the half dose and the maximum tolerable dose when the illuminated area is a few microns in size or smaller, effects that grow with increasing X-ray energy. This can increase maximum tolerable doses in the illuminated volume for micrometer size beams by factors of order 2, but leads to damage outside the beam footprint if the crystal is larger than the beam.

At room temperature, the half-dose $D_{1/2}$ for data collection to a resolution of 1.5-2 Å is roughly 200 kGy for many protein crystals including “standards”, but there is strong evidence that some protein crystals – typically with high solvent contents and weak packing interactions –have half-doses as much as 50 times smaller at equal resolution. The half-dose and maximum tolerable dose at room temperature increase gradually for dose rates above ~5 MGy/s, and are 1.5-2 times larger at dose rates of ~40 MGy/s. This apparent increase in maximum tolerable dose is due to the reduction in irradiation / data collection time to a given dose, which allows slower radiation damage processes to be outrun and data to be collected before the full extent of radiation damage due to the delivered dose has manifested. Half-doses and maximum tolerable doses decrease with increasing temperature, with the rate of increase being larger above the protein-solvent glass transition near 200 K, indicating the important role of radical diffusion and structural relaxation processes that are strongly inhibited at lower temperatures in generating damage.

At all temperatures, the manifestation of radiation damage in diffraction from a given crystal depends strongly on the pattern of crystal irradiation. Ideally (as in the first measurement of half-doses by Teng and Moffat), a crystal is illuminated uniformly throughout its volume using an X-ray beam that is larger than the crystal and whose intensity profile is flat over the crystal area. Instead, crystals are typically illuminated by beams with Gaussian-like profiles and with FWHM values smaller than the lateral crystal dimensions, and crystals may be rotated or rastered within the beam during data collection. Dose and damage are thus spatially varying within the crystal, measured diffraction from the volume illuminated at a given time reflects a range of different damage states, and can lead to nonlinear variation of apparent damage state with dose. These effects of spatially non-uniform irradiation must be carefully considered when evaluating half-doses and maximum tolerable doses.

For additional details, see (Warkentin & Thorne, 2010; Warkentin *et al.*, 2017; Atakisi *et al.*, 2019; Warkentin *et al.*, 2013) and references and Supporting Information contained therein.

S2.2. Dose rates in synchrotron data collection

Warkentin et al. (Warkentin *et al.*, 2017) carefully analyzed doses delivered by gaussian profile X-ray beams to crystals larger than the beam FWHM. Experiments performed at materials science beamline 7-ID at the Advanced Photon Source in 2013 used a 10 keV X-ray beam with a flux of 1.2×10^{12} ph/s that was focused to a spot with a FWHM of 2.5 μm (v) × 5.1 μm (h), with all beam parameters measured immediately prior to diffraction measurements. For a 2D gaussian, half of the total flux is contained within the FWHM. Consequently, the average flux density within the FWHM was 6.1×10^{10} ph/s/μm^2. For lysozyme and thaumatin, respectively, the average doses within the FWHM, calculated using RADDOSE-3D (and verified by manual calculation using NIST tables for atomic absorption coefficients and the known sequence and atomic composition) were 33 and 36 MGy/s. Peak dose rates (at beam center) were 45 and 49 MGy/s. This gives a conversion factor between dose rate and flux

density of ~ 6 MGy/s per 1 × 10^{10} ph/s/μm^2 average within the FWHM – comparable to the value given by Holton (Holton, 2009) for a protein with no metal atoms. The precise value depends on the composition of a crystal's unit cell. The conversion factor roughly varies as the square of the X-ray wavelength λ.

The FMX beamline at NSLS-II can deliver a flux of ~3.5 × 10^{12} ph/s in either a 10 μm or ~ 1 μm × 1.5 μm FWHM beam, corresponding to average flux densities within the FWHM of 2.2 × 10^{10} ph/s/μm^2 and 1.5 × 10^{12} ph/s/μm^2. The corresponding average dose rates for 10 keV photons within the FWHM are then ~12.3 MGy/s and ~815 MGy/s, respectively. Newer and upgraded sources such as ESRF-EBS (ESRF Extremely Brilliant Source) allow focusing of modestly larger monochromatic photon fluxes into beams with FWHM of ~0.5 μm. Using multilayer optics with an energy bandpass of ~3%, beamline ID29-EBSL8 delivers photon fluxes of ~1 × 10^{15} ph/s in a 0.4 μm × 0.6 μm spot size, corresponding to an average flux density within the FWHM of 2.7 × 10^{15} ph/s/μm^2. At 10 keV, this corresponds to an average dose rate within the FWHM of 1.6 TGy/s. The time to deliver a dose of 200 kGy, the nominal (low dose rate) room temperature limit, is then ~0.1 μs.

S2.3. Doses in data collection at X-ray free-electron laser (XFEL) sources

XFELs deliver X-rays in ultra-intense pulses tens of femtoseconds long. With such short pulses, diffraction is complete before atomic displacements associated with energy deposition in the sample become appreciable.

The MFX beamline at the LCLS delivers ~ 1 × 10^{12} ph/pulse in a spot size of 3.7 μm FWHM (Sierra *et al.*, 2019). This corresponds to ~4.7 × 10^{10} ph/μm^2/pulse and to a dose of ~25 MGy/pulse, both averaged over the FWHM. The CXI beamline at LCLS delivers ~ 1 × 10^{12} ph/pulse in a spot of 1.3 μm FWHM (Gati *et al.*, 2017). This corresponds to ~3.8 × 10^{11} ph/μm^2/pulse and to a dose of ~100 MGy/pulse, both averaged over the FWHM. (Gati et al. claim a maximum dose of 1.3 GGy/crystal, but it is not clear how this value was determined; a larger dose per pulse could be due to a large heavy atom concentration in the crystals.) Crystallography beamlines at other XFEL sources give comparable or small doses/pulse.

References

Atakisi, H., Conger, L., Moreau, D. W. & Thorne, R. E. (2019). *IUCrJ*. **6**, 1040–1053.

Gati, C., Oberthuer, D., Yefanov, O., Bunker, R. D., Stellato, F., Chiu, E., Yeh, S. M., Aquila, A., Basu, S., Bean, R., Beyerlein, K. R., Botha, S., Boutet, S., DePonte, D. P., Doak, R. B., Fromme, R., Galli, L., Grotjohann, I., James, D. R., Kupitz, C., Lomb, L., Messerschmidt, M., Nass, K., Rendek, K., Shoeman, R. L., Wang, D., Weierstall, U., White, T. A., Williams, G. J., Zatsepin, N. A., Fromme, P., Spence, J. C. H., Goldie, K. N., Jehle, J. A., Metcalf, P., Barty, A. & Chapman, H. N. (2017). *Proc. Natl. Acad. Sci.* . **114**, 2247–2252.

Holton, J. M. (2009). *J. Synch. Rad.* **16**, 133–142.

Sierra, R. G., Batyuk, A., Sun, Z., Aquila, A., Hunter, M. S., Lane, T. J., Liang, M., Yoon, C. H., Alonso-Mori, R., Armenta, R., Castagna, J. C., Hollenbeck, M., Osier, T. O., Hayes, M., Aldrich, J., Curtis, R., Koglin, J. E., Rendahl, T., Rodriguez, E., Carbajo, S., Guillet, S., Paul, R., Hart, P., Nakahara, K., Carini, G., Demirci, H., Dao, E. H., Hayes, B. M., Rao, Y. P., Chollet, M., Feng, Y., Fuller, F. D., Kupitz, C., Sato, T., Seaberg, M. H., Song, S., Van Driel, T. B., Yavas, H., Zhu, D., Cohen, A. E., Wakatsuki, S. & Boutet, S. (2019). *J. Sync. Rad.* **26**, 346–357.

Warkentin, M. A., Atakisi, H., Hopkins, J. B., Walko, D. & Thorne, R. E. (2017). *IUCrJ*. **4**, 785–794.

Warkentin, M., Hopkins, J. B., Badeau, R., Mulichak, A. M., Keefe, L. J. & Thorne, R. E. (2013). *J. Synch. Rad.* **20**, 7–13.

Warkentin, M. & Thorne, R. E. (2010). *Acta Cryst. D*. **66**, 1092–1100.

Supporting Figures

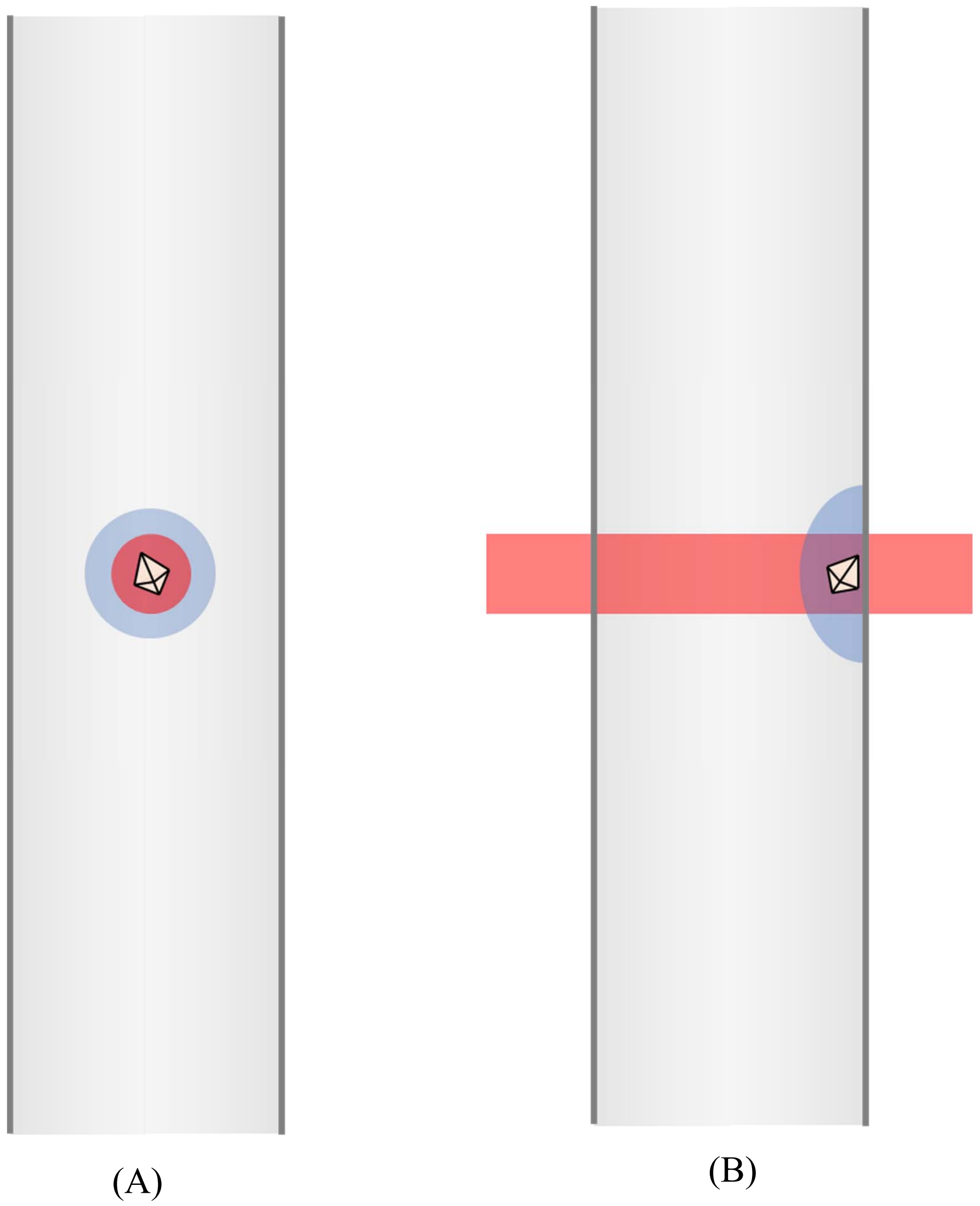

Figure S1 ***Capillary mounting.*** Views (A) along and (B) perpendicular to the incident X-ray beam (red) of a crystal in a glass capillary, held to the capillary wall by a small amount of mother liquor/liquid. Home sources and first and second-generation synchrotron light sources in use prior to the widespread adoption of cryocrystallographic methods delivered large unfocused X-ray beams that were slit down to ~100-200 μm in size, comparable to or larger than the size of crystals, and provided nearly uniform X-ray intensity over the illuminated area. The glass capillary can be replaced by a polymer capillary, and the crystal can be held on a standard crystallography loop/mount inserted into the capillary rather than on the capillary wall, to improve crystal imaging, simplify crystal alignment in the X-ray beam, reduce X-ray background, and reduce crystal slippage. In this approach, X-ray data can be collected from nearly every crystal and over a full 360° of oscillation.

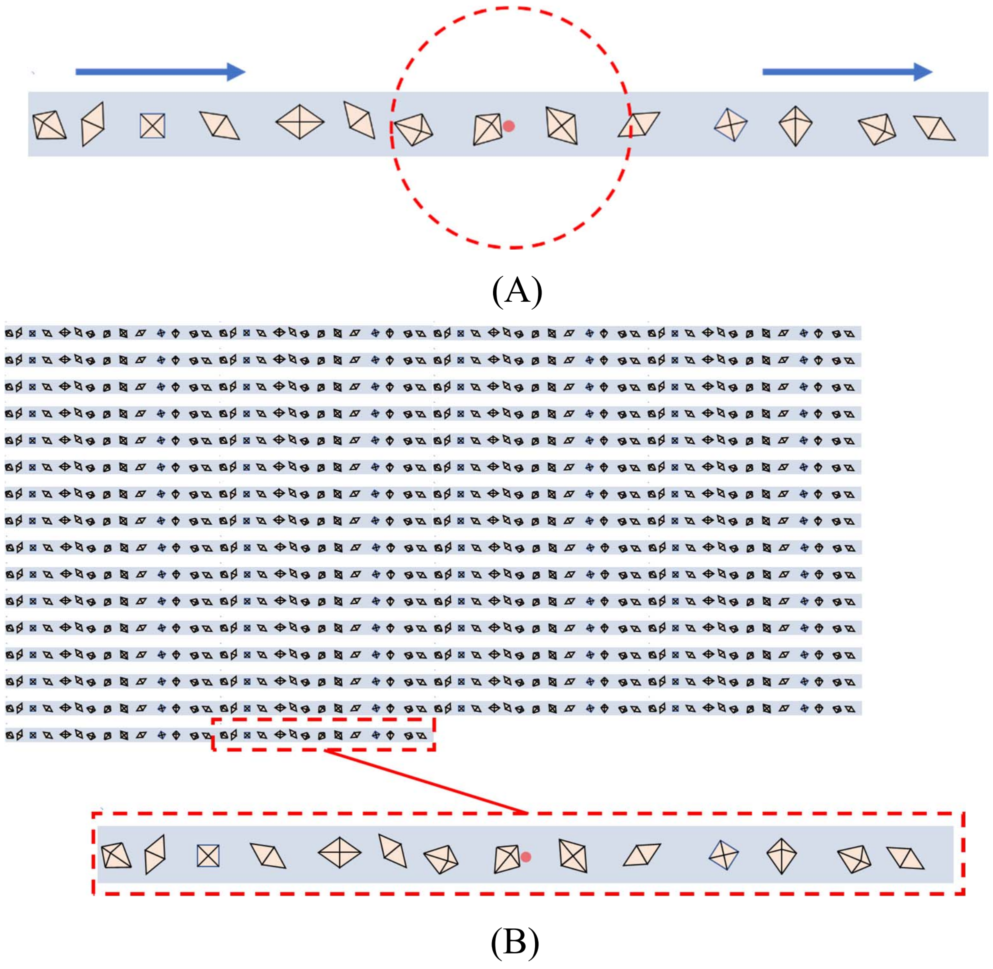

Figure S2 ***Liquid jets.*** (A) A liquid jet moving at 20-50 m/s and with a diameter of ~8 µm carries crystals into an X-ray beam of size ~2 µm (1-3 µm), typically produced by an XFEL source. A region of size ~30 µm around each pulse is disrupted by the X-ray energy deposited (dotted red line). XFELs deliver extremely intense, extremely short X-ray pulses, but only a modest number of pulses per second. The early operating mode of the LCLS produced 100 pulses per second, so the liquid jet flowed ~(0.01 s) × ~30 m/s = 30 cm for each pulse, with only a ~2 µm area within that entire length being illuminated by X-rays. The odds of a crystal being in that 2 µm area – the hit rate – were typically less than 1%. The European XFEL can produce "trains" containing up to 2700 pulses each, at an intra-train rate of up to 4.5 MHz, at a rate of 10 Hz. For a 50 m/s jet and a pulse repetition rate within a train of 1 MHz, the sample jet travels a safe 50 µm between pulses within a train. (B) Assuming (1/4.5) × 2700 = 600 pulses per train, the average pulse separation – including the time between trains – is 1/(600 pulses/train × 10 trains/s) ~ 167 µs, during which the jet travels a distance of ~8.3 mm. Only one ~ 2 µm diameter spot within that entire length will see X-rays, and the probability of a crystal being present at the spot location is <<1. As result, the total volume of crystals required for structure determination is many orders of magnitude larger than is required using conventional methods.

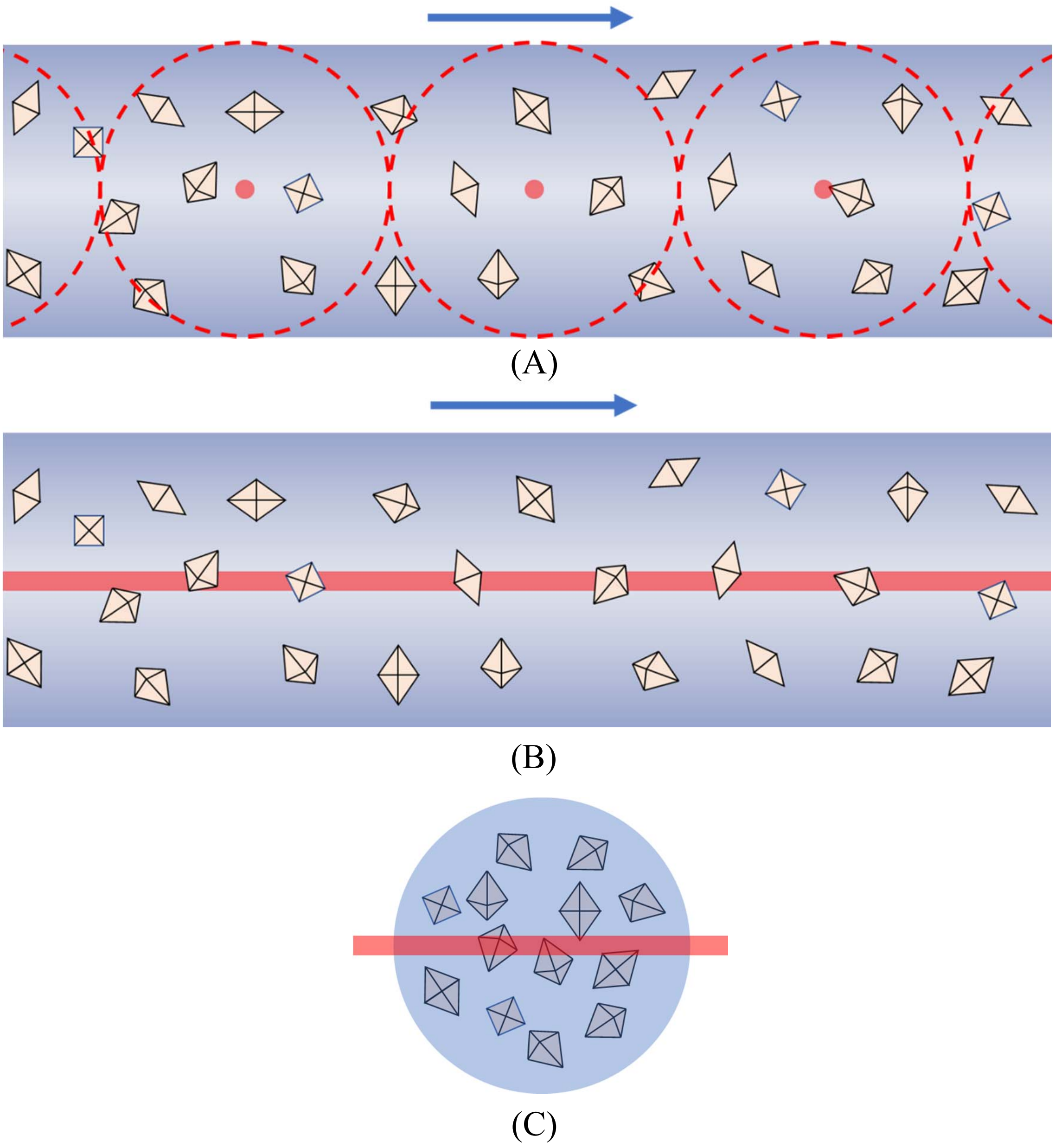

Figure S3 ***High viscosity extrusion (HVE) / LCP extrusion.*** One way to reduce sample consumption at XFELs is to slow the flow of the crystal-containing medium past the X-ray interaction point, so that the distance travelled between X-ray pulses is comparable to the length of crystal-containing medium perturbed by the energy deposited by each pulse. With a pulse rep rate of ~ 100 Hz (at the LCLS) and a 30 µm separation, the required flow rate is of order 3 mm/s, which can be achieved in a controlled way using suitably viscous media and syringe pumps. Typical extruded diameters are ~30 µm. (A) Red dots indicate positions that are exposed to the pulsed, ~ 2 µm diameter X-ray beam as the sample flows through the beam axis and dashed red lines indicate the regions perturbed by each pulse. (B) The same approach can be used to deliver large numbers of crystals at synchrotron sources, where a continuous stripe of sample is illuminated as the sample flows through the beam axis. (C) Axial view of the extruded stream.

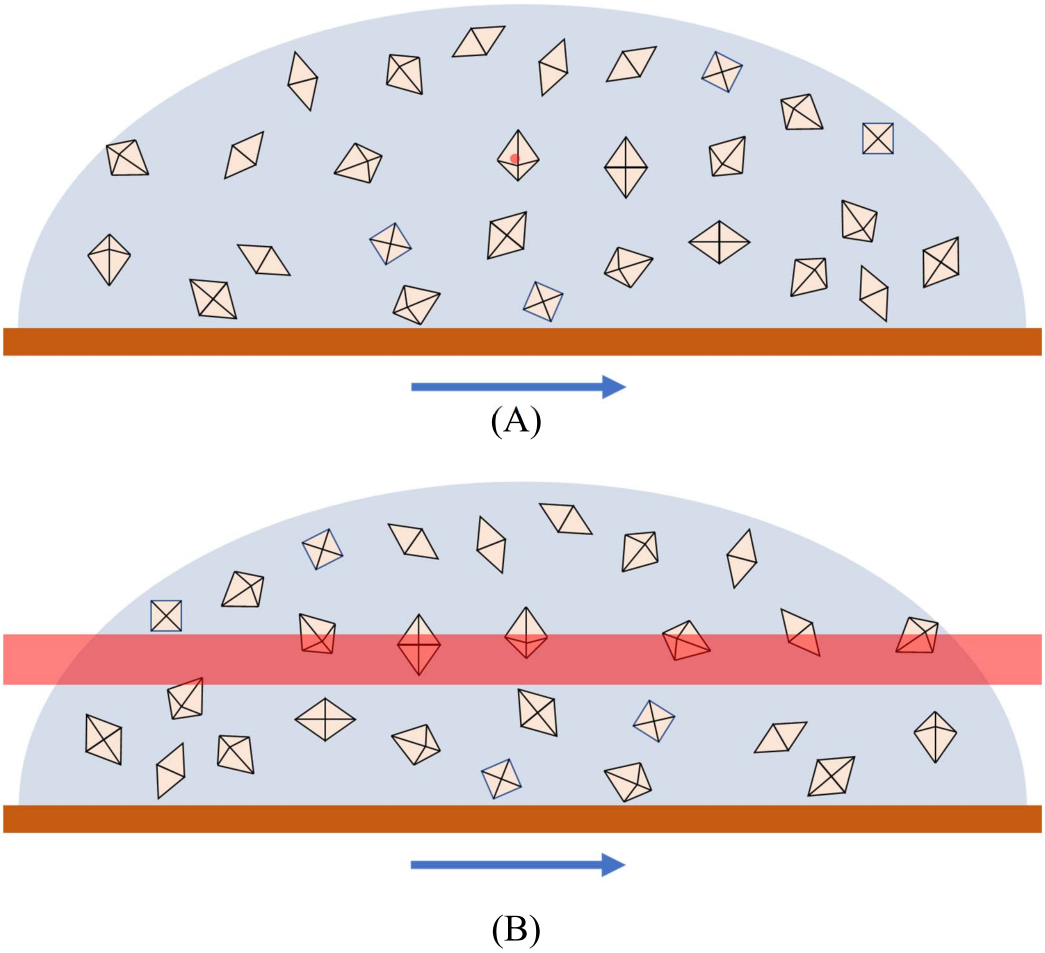

Figure S4 ***Drop-on-tape delivery.*** Another way to reduce sample consumption at XFELs is to deposit crystal-containing drops on a moving polymer tape (a "conveyor belt") that carries the drop through the X-ray beam at a speed matched to the X-ray pulse rate. The standard configuration has the X-ray beam directed parallel to rather than perpendicular to the tape and passing through a crystal-containing drop residing on the tape. Drop-on-demand dispensing systems with tips that can pass crystals in the size ranges of interest deliver minimum drop volumes of a few pL, corresponding to drop diameters of ~250 µm, much larger than the diameter of liquid jets or extruded streams. The chance of the X-ray beam hitting a crystal within that much larger diameter is greater, but background scatter is increased by the same factor. In (A), a 2 µm diameter XFEL pulse (red circle) strikes a 1-2 pL (~200 µm diameter) crystal-containing drop. In (B), a 10 µm diameter synchrotron beam illuminates the region shown as the drop translates through the beam.

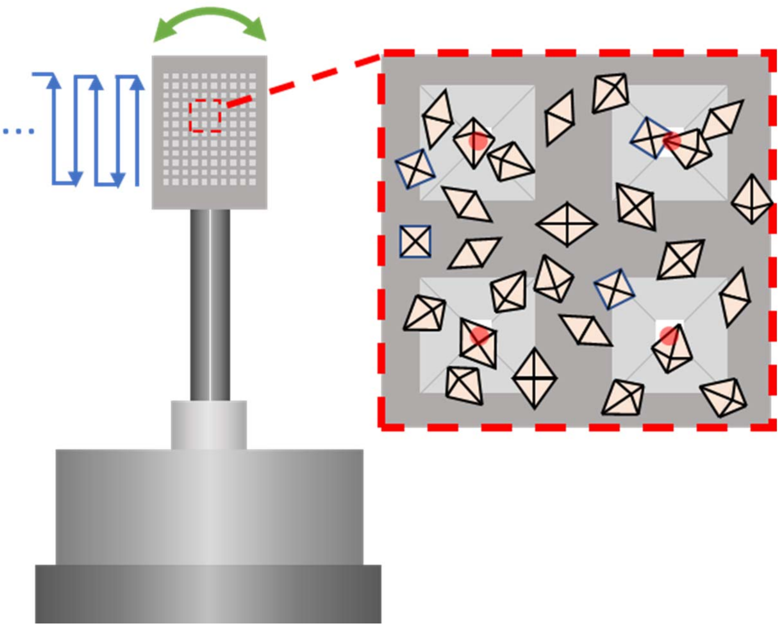

Figure S5 ***Step-and-shoot fixed-target arrays.*** In this "fixed-target" approach, crystals and solution are deposited on a support (usually of silicon) containing an array of wells with through-holes. Liquid is removed through the holes by suction or blotting from the backside of the array, and some crystals are drawn down to the bottom of the wells where they may cover the holes. The X-ray beam is directed through the hole in the bottom of each well to interrogate any crystals present there. Then the ("fixed target") array is translated to place the hole in the next well in the beam path. Holes are typically 5 to 25 µm, depending on the size of crystals to be trapped. Only a small fraction of the crystalline material present on the array is typically in the holes and accessible to the X-ray beam (indicated by the red circles). When using synchrotron sources, the support can be oscillated through a modest angular range (green arrows) during data collection to improve indexing.

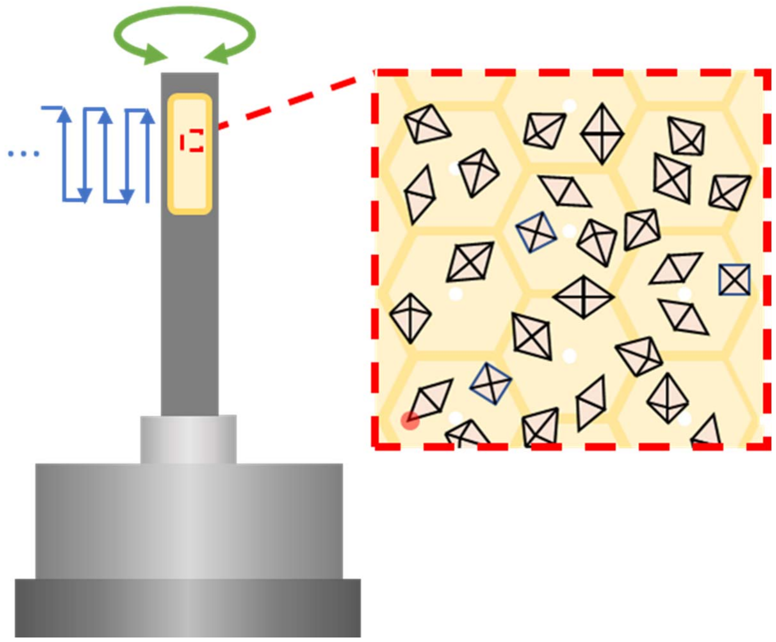

Figure S6 ***Full raster fixed-target arrays.*** In this "fixed-target" approach, the crystals are supported on a thin, X-ray transparent film that is either unsupported or that spans an aperture in a rigid frame. X-ray data can then be collected from all crystals, not just those near holes, by rastering the array in the X-ray beam (red circle). Through holes allow removal of excess liquid, and film patterning can help guide crystals to particular regions or fix them in place. When using synchrotron sources, the array can be oscillated through >240°, allowing collection of complete data sets from individual crystals when crystal size and order permit doing so.

(A)

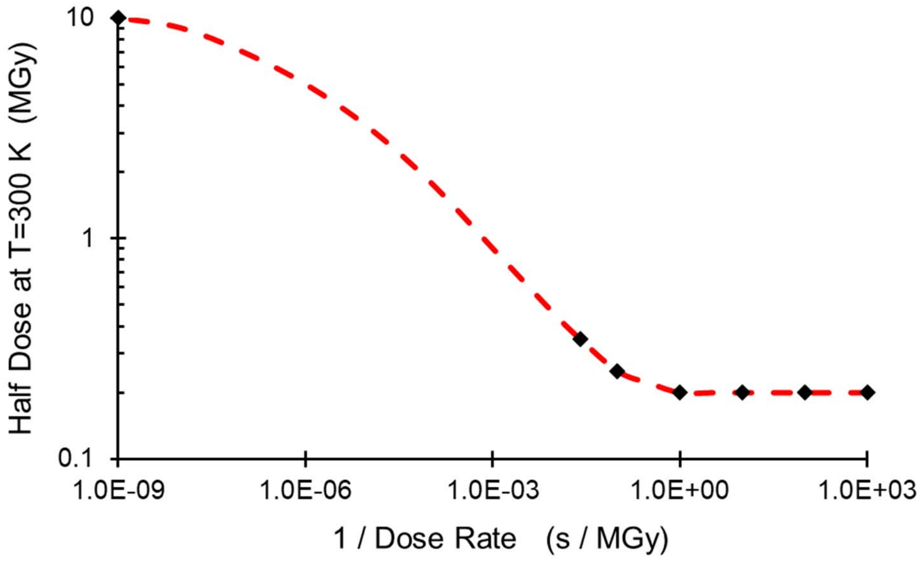

(B)

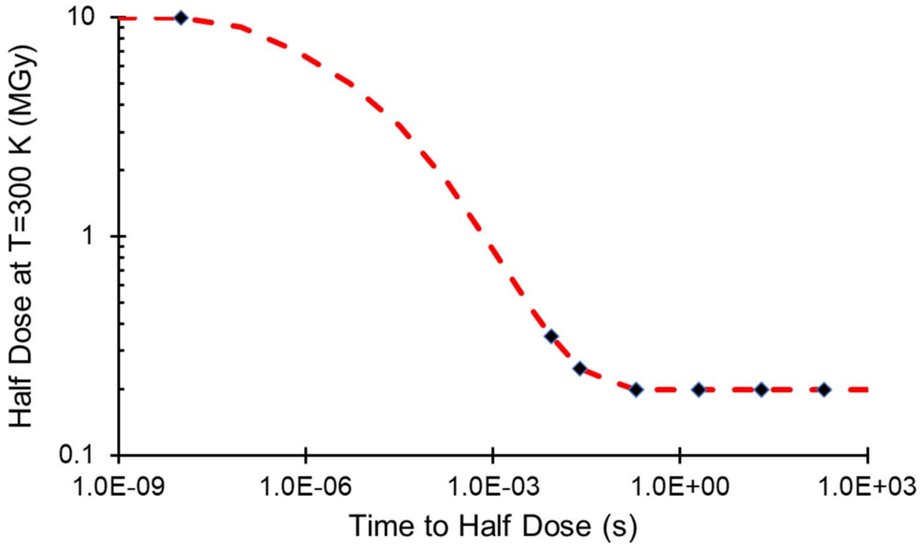

Figure S7 Experiment-based (solid points) and extrapolated (dashed line) variation of the half-dose with (A) inverse dose rate in seconds per MGy and (B) exposure time to the half-dose in seconds, at room temperature and assuming a resolution limit of roughly 1.5 Å. The half-dose is the dose at which the integrated intensity in Bragg reflections falls to half its zero-dose limit, and roughly corresponds to the maximum tolerable dose without excessive resolution loss. The timescales for the diffusive processes responsible for excess radiation sensitivity at room temperature relative to cryogenic temperatures are likely ns and longer, and so the cryogenic temperature half dose of ~10 MGy should be observed at room temperature for exposures shorter than these timescales. Experiment-based points are from Warkentin et al., 2017 for lysozyme and thaumatin crystals. Anecdotal evidence indicates that some biomolecular crystals may be as much as 50 times more sensitive at room temperature than lysozyme but are comparably radiation sensitive at cryogenic temperatures. The half-doses in (A) and (B) at large inverse dose rates / for long exposure times may then be upper bounds.

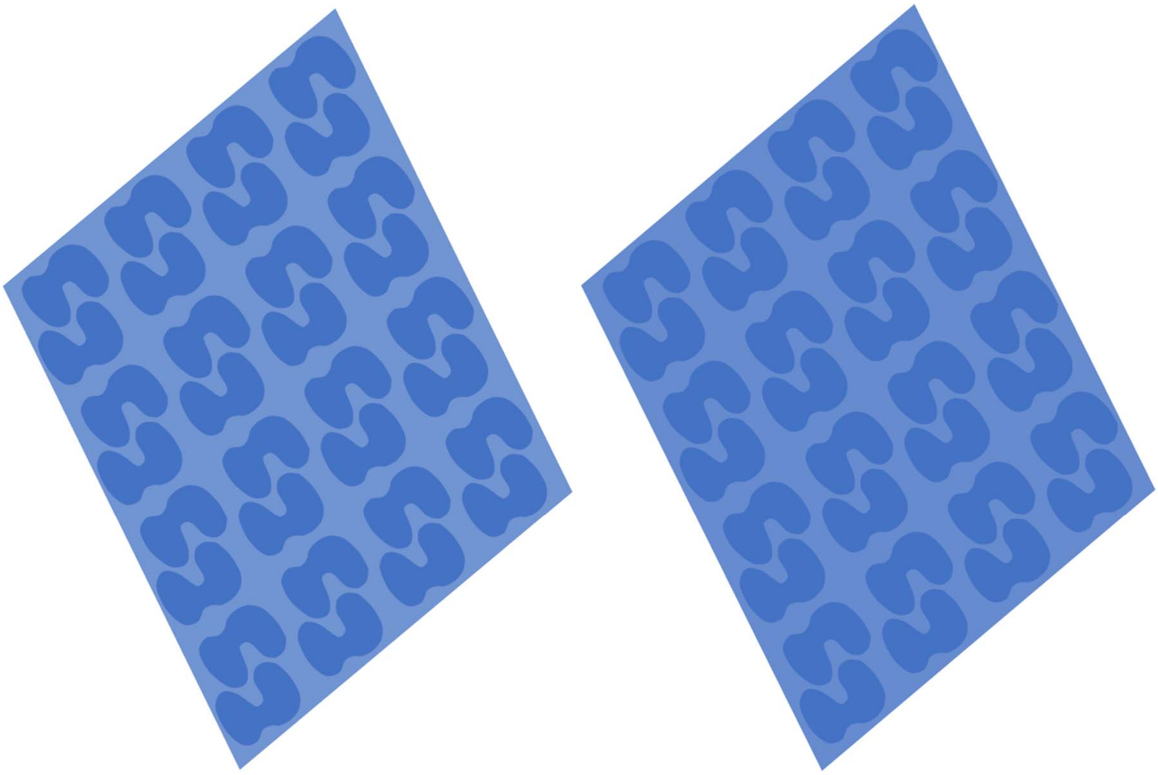

(A) (B)

Figure S8 The integrated intensity within each Bragg diffraction peak is proportional to the electron density difference (or "contrast") between the protein molecules and the internal crystal solvent. At room temperature, protein has an average electron density of ~0.43 e^- $Å^{-3}$ and water has an electron density of 0.31 e^- $Å^{-3}$. Solutions of most cryoprotective agents used to prevent ice formation in cryocrystallography have higher electron densities than pure water and so reduce electron density contrast relative to what can be obtained in cryoprotectant-free room-temperature crystallography. In (A), the shading reflects the electron density difference between protein and water. In (B), the shading reflects the electron density difference between protein and a 30% w/w glycerol solution. The Bragg peak intensities corresponding to (B) are ~37% weaker than in (A).

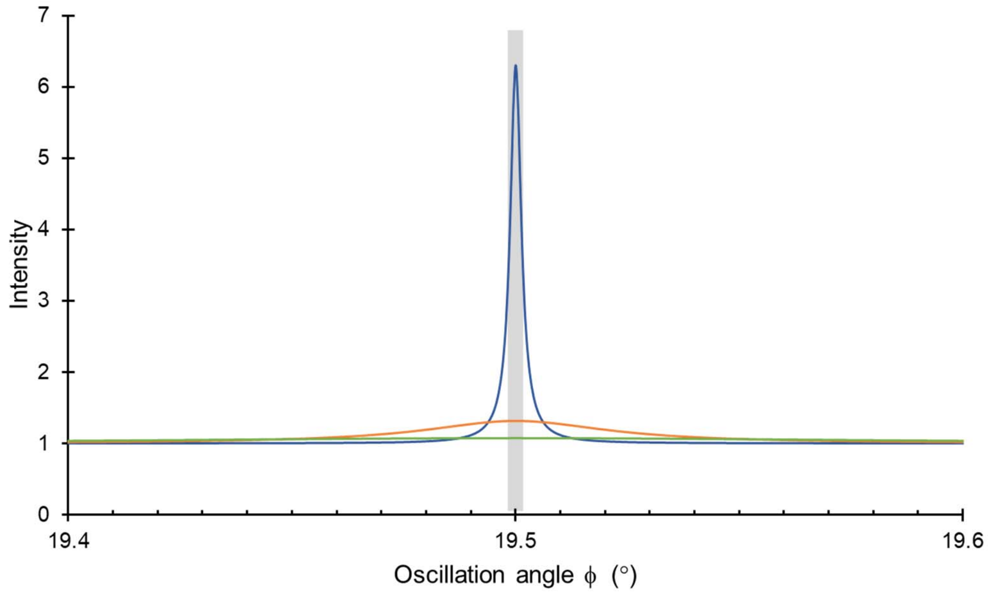

Figure S9 Effect of crystal mosaicity and incident beam divergence on recorded Bragg peak intensities calculated using a simple model. A constant background intensity of 1 is assumed. The blue and orange curves show the recorded intensity versus oscillation angle ϕ for a crystal at room temperature with a mosaicity of 0.003° FWHM, using a non-divergent X-ray beam and a beam with a 0.05° divergence angle, respectively. The green curve shows the intensity for a crystal at 100 K with a mosaicity of 0.2° recorded using a beam with 0.05° divergence. If each diffraction frame records 0.2° (the full plotted ϕ range), then the background-subtracted integrated intensity normalized by the uncertainty σ (estimated as the square-root of the total integrated peak intensity) is ~2.3 for the room temperature crystal and 1.2 for the 100 K crystal. If each diffraction frame for room temperature data collection instead records 0.003° of oscillation (the FWHM of the peak, corresponding to the shaded interval), then the intensity/uncertainty for the room temperature peak improves to 4.7. Thermal diffuse background will be larger at room temperature, and background due to static crystal disorder will be larger at cryogenic temperature.
